# Supplementary material for: New Methods Used in Pharmacokinetics and Therapeutic Monitoring of the First and Newer Generations of Antiepileptic Drugs (AEDs)
Source: Molecules. 2020 Nov 2;25(21):5083. doi: 10.3390/molecules25215083 (PMC7663638; doi:10.3390/molecules25215083)
Supplement: Supplementary file 1 [file molecules-25-05083-s001.pdf]

Table 1. Characteristics of AEDs.

Drug	Generation	Drug Approval Date	Chemical Classification/Structure, Molecular Mass [g/mol]	Mechanism of Action	Pharmacokinetics/Metabolism	Therapeutic Range [mg/L]	Drug-Drug Interactions	Ref.
Brivaracetam (BRV)	III	2016 (USA)	((2S)-2-((4R)-2-oxo-4-propylpyrrolidinyl)butanamide $C_{11}H_{20}N_2O_2$ 212.288	acting on SV2A, through the synaptic GABA release, sodium channels inhibitor	bioavailability ~100%, T_{max} 1 h, $T_{0.5}$ 7–8 h, <20% bound to plasma proteins, metabolized by hydrolysis and oxidation, >95% excreted in urine with <10% of compound unchanged	0.2–2.0	baclofen with BRV increase adverse effect, CBZ may increase the thyroid function activities of BRV and VPA decreases.	[94,95,97]
Carbamazepine (CBZ)	I	1965 (UK) 1968 (USA)	Benzo(b)(1)benzazepine-11-carboxamide $C_{15}H_{12}N_2O$ 236.269	inhibition of the voltage-gated sodium channel (VGSC)	bioavailability 75–85%, T_{max} 19 ± 7 h, $T_{0.5}$ 35 h protein binding 65–85%, hepatic metabolism via CYP3A4/5 and in a lesser extent CYP 2C8, main active epoxide metabolite—(CBZE), excreted in the urine as N-glucuronide (15%)	4–12	PHT, PB, PRM increase CBZ clearance and reduce its half-life. VPA inhibits epoxide hydrolase, and CYP 3A4 inhibitors increase CBZ and CBZE blood levels	[10,29,43,69,189,190]
Cenobamate (CNB)	new	2019 (USA)	((1*R*)-1-(2-chlorophenyl)-2-(tetrazol-2-yl)ethyl) carbamate $C_{10}H_{10}ClN_5O_2$ 267.670	positively modulates γ-$GABA_A$ and inhibits voltage gated sodium channels	bioavailability 88%, T_{max} 1–4 h, $T_{0.5}$ 50–60 h, 60% protein bound in plasma, extensively metabolised in the liver via glucuronidation and oxidation	NA	CNB inhibits CYP2C19: decreases PHT and PHB; induces CYP3A4: decreases LTG; PH causes decreases CNB	[1,2,185–187]
Clobazam (CLB)	I	1975 (anxiolytic) 1984 (anticonvulsant) 2011 (USA)	7-chloro-1-methyl-5-phenyl-1,5-benzodiazepine-2,4-dione $C_{16}H_{13}ClN_2O_2$ 300.740	inhibitory effect of GABA on neuronal excitability by increasing neuronal membrane permeability to chloride ions	bioavailability ~100%, T_{max} 1–4 h, $T_{0.5}$ 10–30 h plasma protein-binding 80–90%, main metabolite -N-desmethylclobazam (N-CLB), hepatic elimination (98%), excreted renally	0.03–0.3	CBZ, PHT, PB induced CLB metabolism and increased N-CLB levels, cimetidine (an inhibitor of several CYPs (CYP 2C19) increased N-CLB concentration	[29,82,191]
Eslicarbazepine Acetate (ESL)	III	2010 (Europe) 2013 (USA)	5-oxo-6*H*-benzo(b)(1)benzazepine-11-carboxamide $C_{15}H_{12}N_2O_2$ 296.326	competitive inhibitor of VGSC	bioavailability > 90%, <40% protein binding, T_{max} 1–4 h, $T_{0.5}$ 13–2 0h, main metabolite: S-licarbazepine, renal elimination (>90%), mainly as ESL (52%) and ESL-glucuronide (41%)	3–26	interaction with other AEDs: PHT, PB, CBZ, oral contraceptive (ethinylestradiol and levonorgestrel) and simvastatin	[4,29,45,79,80,193]

Table 1. *Cont.*

Ethosuximide (ESM)	I	1960 (USA)	3-ethyl-3-methylpyrrolidine-2,5-dione $C_7H_{11}NO_2$ 141.168	voltage-gated T-type calcium channels	bioavailability 90–100%, T_{max} 1–7 h, $T_{0.5}$ 25–60 h, protein binding unknown, hepatic extensive metabolism by CYP3A4 and CYP2E1, clearance may be saturable (nonlinear) at higher doses, renal elimination 20%	40–100	CBZ, PHB, PHT, PRM, and rifampicine increase elimination, in consequence, decrease the plasma level of ESM. STP, isoniazid inhibits metabolism of ESM and increase ESM plasma levels	[27,29,38]
Felbamate (FBM)	II	1993 (USA)	2-phenyl-1,3-propanediol dicarbamate $C_{11}H_{14}N_2O_4$ 238.239	weak inhibitor on GABA- and benzodiazepine receptor, antagonist at the strychnine-insensitive glycine recognition site of NMDA receptor-ionophore complex	bioavailability > 90%, T_{max} 1–4 h, $T_{0.5}$ 20–23h , protein binding 20–36%, hepatic metabolism (50% to inactive products)	30–60	CBZ, PHT, PB increase whereas VPA decreases its metabolism	[119,120]
Gabapentin (GBP)	II	1993 (USA)	1-(aminomethyl)cyclohexaneacetic acid $C_9H_{17}NO_2$ 171.237	inhibits α2-δ subunit of voltage-gated calcium channels	non-linear pharmacokinetics, bioavailability < 60%, T_{max} 2–3 h, $T_{0.5}$ 5–9 h, not metabolized, not protein bound, renal elimination	2–20	hydrocodone, cimetidine, morphine, and naproxen increase, whereas antacids decrease its concentration	[138,193]
Lacosamide (LCM)	III	2008 (Europe) 2009 (USA)	(R)-2-acetamido-N-benzyl-3-methoxypropionamidel $C_{13}H_{18}N_2O_3$ 250.294	inhibits sodium channel, selective inhibitor of depolarized neurons, binds to collapsin response mediator protein-2 (CRMP-2)	bioavailability~100%, T_{max} 1–4 h, $T_{0.5}$~13h, <15% bound to plasma proteins, as a CYP2C19 substrate, excreted unchanged (40%) with its inactive O-desmethyl metabolite (30%)	3–10	strong interaction with selective serotonin 5-HT3 receptor antagonist (dolasetron) or antiretroviral protease inhibitor (saquinavir). Interact with beta-blockers like metoprolol and diltiazem or verapamil	[161,162,194]
Lamotrigine (LTG)	II	1994 (USA)	3,5-diamino-6-(2,3-dichlorophenyl)-as-triazine $C_9H_7Cl_2N_5$ 256.091	inhibits a voltage-sensitive sodium channels, modulates the release of aspartate and glutamate	bioavailability > 95%, T_{max} 1–3 h, $T_{0.5}$ 15–35 h, protein binding 66%, extensively metabolized via glucuronidation	2.5–15	LTG increases VPA	[29,195]
Levetiracetam (LEV)	II	1999 (USA)	(-)-(S)-α-ethyl-2-oxo-1-pyrrolidine acetamide $C_8H_{14}N_2O_2$ 170.212	acting on SV2A, indirectly GABAergic neurotransmiter and ionic currents modulater, in vitro inhibitor of N-type calcium channels	bioavailability ~100%, T_{max} 1.3 h, $T_{0.5}$~6–8 h, enzymatic hydrolysis to inactive carboxylic acid metabolite (L057), 66% excreted in the urine as unchanged drug	5–41	LEV interact with alcohol, antihistamines, antipsychotics or benzodiazepines, SSRI and other seizure drugs like CBZ, VPA or ZNS	[96,98]

Table 1. *Cont.*

Oxcarbazepine (OXC)	II	2000 (USA)	10,11-dihydro-10-oxo-5H-dibenz (b,f) azepine-5-carboxamide $C_{15}H_{12}N_2O_2$ –252.269	inhibition of the voltage-gated sodium channel (VGSC)	bioavailability >9 5%, T_{max} 1–3 h, $T_{0.5}$ 1–5 h, 60% protein binding, main active metabolite - 10,11-dihydro-10-hydroxy-carbazepine, clearance route: hepatic (50%) and renal (50%)	10–35	dose dependent effect of the metabolism of dihydropyridine antagonists, oral contraceptives and some antiepileptic drugs (CBZ)	[29,43,68,191]
Perampanel (PER)	III	2012 (USA)	2-(2-oxo-1-phenyl-5-pyridin-2-ylpyridin-3-yl)benzonitrile $C_{23}H_{15}N_3O$ 349.393	selective, noncompetitive AMPA (α-amino-3-hydroxy-5-methyl-4-isoxazolepropionic acid) type glutamate receptor antagonist	bioavailability: ~100%, T_{max} 0.5–2 h, $T_{0.5}$~105 h 95% plasma protein binding, extensive hepatic metabolism (>90%), excreted as oxidative and conjugated metabolites with urine and feces	0.1–1.0	PER interacts with other AEDs (OXC, TPM, VPA, CBZ, PHT), alcohol, benzodiazepines, narcotics, barbiturates, antihistamines	[4,29,44,45,168,169,191]
Phenobarbital (PHB)	I	1912 (Germany)	5-ethyl-5-phenyl barbituric acid $C_{12}H_{12}N_2O_3$ 232.235	interaction with $GABA_A$ and increase in chloride ions in neuron and finally reduce neuronal excitability	bioavailability > 95%, T_{max} 2–3 h, $T_{0.5}$ 30–173 h in adults; 50 h in children, 20–45% plasma protein binding, extensively (>70%) metabolized by isoenzymes of cytochromes CYP2C9, CYP2C19, and CYP2E1	10–40	interactions are results of inducing effect of phenobarbital on CYP1A2, CYP3A6, CYP2B, CYP2C, CYP3A4 and UTGs and is observed decreased level of AEDs and beta-blockers, calcium channel blockers, digoxin, hormonal contraceptives statins: lovastatin, simvastatin, cerivastatin, and atorvastatin, vitamin	[14–18]
Phenytoin (PHT) and Fosphenytoin (FOS)	I	1908 (Germany) 1953 (USA 1996 (USA)	5,5′-diphenylhydantoin $C_{15}H_{12}N_2O_2$ 252.268 (3-phosphoryloxymethyl)phenytoin $C_{16}H_{15}N_2O_6P$ 362.274	modulation of voltage-gated sodium channels-enhances rapid inactivation of sodium channels	hepatic metabolism in 98%, by the isoenzyme CYP2C9 and CYP2C9. Biological half—life elimination is generally in the range 7–42 h and can be extended because of saturable pharmacokinetics, $T_{0.5}$ 15 min to convert FOS to PHT. Phenytoin is an inducer of CYP3A4, CYP2C9, CYP2C19, CYP1A2 and UGT	10–20 (total) 1–2 (free)	decreased level of CBZ and CBZE, clonazepam, FBM, LTG, OXC, PER, PRM, RFM, TPM, VPA, ZNS, TGB, decreased level of digoxin, hormonal contraceptive, ibrutinib, nilotinib, proton pump inhibitors, quinidine, sirolimus, tacrolimus, statins, telaprevir, vitamin D and folic acid	[25–29]

Table 1. *Cont.*

Piracetam (PIR)	I	1970 (Europe)	(2-oxo-pyrrolidin-1-yl)-acetamide $C_6H_{10}N_2O_2$ 142.158	nootropic modulator of cerebral function, positive allosteric regulator of AMPA (α-amino-3-hydroxy-5-methyl-4-isoxazolepropionic acid) receptor	bioavailability ~100%, T_{max} 1–1.5 h, $T_{0.5}$~5 h, not protein-bound, renal elimination as unchanged drug (not known major metabolism)	NA	PIR increases level of CBZ, cisplatine, digoxine, estradiol acetate, lithium, carbonate, procainamid, tiapride, vancomycin	[86]
Pregabalin (PGB)	II	2004 (USA)	(S)-3-(aminomethyl)-5-methylhexanoic acid $C_8H_{17}NO_2$ 159.226	inhibits α2-δ subunit of voltage-gated calcium channels	bioavailability > 90%, T_{max} 1–2 h, $T_{0.5}$ 5–7 h, not metabolized, not protein bound, renal elimination	2–5	GBP and PHT decrease its concentration	[29,138]
Primidone (PRM)	I	1954 (USA)	5-Ethyl-5-phenyl-1,3-diazinane-4,6-dione $C_{12}H_{14}N_2O_2$ 218.252	binds synaptic and extrasynaptic $GABA_A$ receptors	bioavailability 60–100%, $t_{0.5}$ 10–12 h (PEMA), 29–3 6h (PHB), renal excretion 40–60% unchanged, hepatic metabolism CYP2C9, CYP2C19, active metabolites—phenylethylmalonamide (PEMA) and PHB	5–12 (PRM), 15–40 as (PHB)	interactions similar to PHB, are results of inducing effect of phenobarbital on CYP1A2, CYP3A6, CYP2B, CYP2C, CYP3A4 and UTGs	[26,29,35]
Rufinamide (RFM)	III	2007 (Europe) 2008 (U[29,126,196]SA)	1-(2,6-difluorobenzyl)-1H-1,2,3-triazole-4- carboxamide $C_{10}H_8F_2N_4O$ 238.194	sodium channel inhibitor	bioavailability 70–85%, T_{max} 4–6 h, $T_{0.5}$ 6–10 h, ~40% bound to plasma proteins, no active metabolites, excreted renally (66% as CGP 47292 metabolite and 2% as unchanged drug)	5–30	RFM as a mild interactor, increase the clearance of oral contraceptives (ethinyl estradiol, norethindrone)	[197,198]
Stiripentol (STP)	III	2018 (USA)	4,4-dimethyl-1-(3,4-(methylendioxy)-phenyl)-1penten-3-ol $C_{14}H_{18}O_3$ 234.295	increases the activity of $GABA_A$ receptors	non-linear pharmacokinetics, bioavailability not available, T_{max} 0.5–2 h, $T_{0.5}$ 4.5–13 h, protein binding 96%, extensive metabolism by the CYP1A2, CYP2C19, and CYP3A4	4–22	inhibits CYP 1A2, 3A4, 2C19, 2D6: increases CBZ, CLB, PHT, PB and VPA	[199–201]
Sulthiame (STM)	I	1950 (Germany)	4(1,1dioxothiazinan-2-yl) benzenesulfon amide $C_{10}H_{14}N_2O_4S_2$ 290.359	membranę—a permeant inhibitor of the enzyme carbonic anhydrase	hydroxylation and renal excretion 30–60%, C_{max} 0.02 and 1.88 mg/L after 50 and 200 mg dose.	5–35	CBZ and PRM, PHT increase the elimination of STM; antacids decrease GI absorption.	[4,38,42]
Tiagabine (TGB)	II	1996 (Denmark) 1997 (USA)	N-(4,4-di(3-methylthien-2-yl)but-3-enyl)nipecotic acid $C_{20}H_{25}NO_2S_2$ 375.548	inhibits GABA reuptake into neurons and glia	Dose-dependent, plasma $T_{0.5}$ 40–90h, whole blood $T_{0.5}$ 253–313 h bioavailability ≤9 0%, T_{max} 0.5–2 h, $T_{0.5}$ 5–9 h, protein binding 98% extensively metabolized by CYP3A4	0.02–0.3	CBZ, PHT, PB increase its clearance, VPA increases free fraction	[29,202]

Table 1. *Cont.*

Topiramate (TPM)	II	1996 (USA)	2,3:4,5-Bis-O-(1-methylethylidene)-beta-D-fructopyranose sulfamate $C_{12}H_{21}NO_8S$ 361.362	Inhibits voltage-dependent sodium and calcium channels, it also inhibits carbonic anhydrase activity. Enhances the inhibitory effect of GABA.	bioavailability >80%, $T_{0,5}$ 20–30 h, metabolism mainly by hydroxylation, hydrolysis glucuronidation, and sulfonation 20–30% metabolized in monotherapy, increase to 50–70% in polytherapy with CBZ and PHT	5–20 2–10	TPM clearance is increased by CBZ, OXC, and PHT, but decreased by lithium, propranolol, amitriptyline	[26,45,113]
Valproic acid (VPA)	II	1967 (France) 1978 (USA)	2-propylpentanoic acid $C_8H_{16}O_2$ 144.211	increases the inhibitory activity of GABA, blocks voltage-gated ion channels, inhibits histone deacetylase	non-linear pharmacokinetic, bioavailability 90–100%, T_{max} 1–7 h, $T_{0.5}$ 9–16 h, protein binding 85–95%, extensive metabolism via β-oxidation glucorunidation and CYP-mediated oxidation	50–100	inhibits CYP2C9, CYP3A4, and epoxide hydrolase: increases CBZ, ESM, LTG, PHB and RFM	[29,126,196]
Vigabatrin (VGB)	III	2003 (Mexico) 2009 (USA)	**4-amino-5-hexenoic acid** $C_6H_{11}NO_2$ 129.157	inhibits GABA-transaminase	bioavailability 60–80%, T_{max} 1–2 h, $T_{0.5}$ 5–8 h, protein binding 17%, not metabolized, renal elimination	0.8–36	FBM increases its elimination	[29,126,203]
Zonisamide (ZNS)	II	1989 (Japan) 2000 (USA) 2005 (Europe)	1,2-benzisoxazole-3-methanesulfonamide 1,2-benzoxazol-3-ylmethanesulfonamide $C_8H_8N_2O_3S$ 212.226	double mechanism of action due to weak enzyme inhibition and modulation of GABA-ergic and glutamate neurotransmission by changing voltage sensitive sodium and calcium channels	bioavailability ≥ 90%, T_{max} 2–5 h, $T_{0.5}$ ~60 h protein binding (40–50%), hepatic metabolism through CYP3A4 (acetylation and reduction, forming N-acetyl zonisamide and 2–sulfamoylacetyl phenol), excreted primarily in urine as parent drug and as the glucuronide of a metabolite	10–40	significantly increases CBZ elimination via induction of CYP3A4, PHT and PB induce ZNS metabolism	[10,29,45,157, 191,192]

Table 2. Recent methods for analysis of AEDs in biological matrices.

Analyzed Drugs	Biological Matrix	Sample Preparation	Method	Column and Mobile Phase	LOQ; Calibration Range	Application	Co-Detected Compounds	Ref
Brivaracetam	plasma	Protein precipitation with acetonitrile	UHPLC-MS/MS m/z: 213.07 → 168.15 (BRV), 210.10 → 175.19 (IS)	Synergi Fusion column (75 m × 2.0 mm, 5 µm) 0.1% formic acid in water/acetonitrile, gradient mode	0.1 mg/L; 0.1–10 mg/L	Therapeutic drug monitoring in epilepsy patients	Brivaracetam-d7 (IS)	**[99]**
Brivaracetam	liver and kidney tissue homogenates, blood (rats)	SPE (off-line)	LC-MS/MS m/z: 214.0 → 168.0 (BRV-AC), 230.0 → 184.0 (BRV-OHAC), 233.0 → 188.0 (IS)	Waters Atlantis T3 C18 (50 mm × 2.1 mm, 5 µm) Water and acetonitrile (99:1, v/v), gradient mode	0.001 mg/L; 0.001–0.2 mg/L	In vitro metabolism assay	BRV metabolites (carboxylic-BRV-AC and hydroxylated-BRV -OHAC); Seletracetam (IS)	**[107]**
Brivaracetam	plasma	LLE with tert-Butyl methyl ether	UHPLC-MS/MS m/z: 213.12 → 237.06 (BRV), 237.06 → 193.25 (IS)	Aquity BEH C18 column (100 mm × 2.1mm, 1.7 µm) acetonitrile:0.1% formic acid in water	0.002 mg/L; 0.002–2 mg/L	Pharmacokinetics study in rats and therapeutic drug monitoring, pharmacokinetic study, forensic analysis	CBZ (IS)	**[102]**
Carbamazepine	urine	Dispersive LLME (a mixture of acetonitrile and urine sample—homogenous solution) with solid sodium chloride)	GC-FID	HP–5 capillary column (30 m × 0.32 mm,0.25 µm) Carrier gas: nitrogen	0.033 mg/L; 0.04–100 mg/L	To determine and detect carbamazepine and phenobarbital in urine	PHB	[58]
Carbamazepine	plasma and urine	Ultrasound—assisted emulsification microextraction (SAEME) with 1-octanol in water	GC-FID	DB5 (25 m × 0.32 mm; 0.25 µm) Carrier gas: helium	1.2 mg/L (plasma); 5.0–500 mg/L (plasma) 0.6 mg/L (urine); 2.5–500 mg/L (urine)	Analysis of carbamazepine in biological samples	None	[46]
Carbamazepine	dried blood spot (DBS)	LLE with acetonitrile and sodium hydroxide (24:1, v/v) followed by derivatization with N-methyl-N-trimethylsilyl-trifluoroacetamide and trimethylchlorosilane	GC-MS m/z: 193 (CBZ), 201 (VPA), 281 (PHT), 267 (IS)	DB5 (30 m × 0.25 mm, 0.25 µm)	0.07 mg/L; 0.5–120 mg/L	TDM 169 patients with epilepsy (PWE) on mono- or polytherapy of CBZ, PHT or/and VPA were included in the analysis	VPA, PHT, 5-(p-methylphenyl)-5-phenylhydantoin (IS)	[63]

Table 2. *Cont.*

Carbamazepine	plasma	Protein precipitation with acetonitrile	HPLC-UV λ=220 nm	Nova-Pak® C18 Mixture of dihydrogen phosphate buffer (pH 6.0)–acetonitrile-2–propanol (63:22:15, v/v/v)	0.07 mg/L; 0.3–15 mg/L	Determination of AED for a large number of a patient sample	CBZE, PHB, PHT	[47]
Carbamazepine	plasma	Protein precipitation with chloroform	HPLC-DAD λ=220 nm	Intersil DS-4 C18 (150 mm × 4.6 mm, 5 µm) acetonitrile-water (50:50, v/v)	0.5 mg/L; 0.05–16 mg/L	Clinical study for monotherapy or polytherapy of carbamazepine	Propyloparaben (IS)	[51]
Carbamazepine	serum	LLE with ethyl acetate	HPLC-DAD λ = 210–400 nm	Bonus-RP (150 mm × 0.46 mm, 5 µm) acetonitrile/K_2HPO_4 buffer solution (45:55)	**0.1** mg/L; 0.1–9.1 mg/L	TDM of CBZ	None	[55]
Carbamazepine	dried saliva spots (DSS)	LLE with methanol and formic acid (pH 5.5) using Whatman TM903 protein saver card	HPLC-DAD λ=210 nm	Zorbax SB-C18 (250 mm × 4.6 mm, 1.8 µm) 35% acetonitrile/65% water-methanol-trimethylamine (75.5:24.2)	0.1 mg/L; 0.1–10 mg/L	Alternative for AEDs blood monitoring	PHT, PHB	[60]
Carbamazepine	plasma	Microextraction by packed sorbent (MEPS)	HPLC-DAD λ=215 nm (CBZ, CBZE, PB, LTG, PHT and LIC) λ=237 nm (OXC) λ=280 nm (IS)	LiChroCART® Purospher Star column C18 (55 mm × 4 mm, 3 µm) acetonitrile (6%), mixture (94%) water-methanol-triethyloamine (73.2:26.5:0.3; v/v/v)	NA; 0.1–15 mg/L	Routine TDM of CBZ, LTG, OXC, PB and PHT	LTG, OXC, PB, PHT, CBZE, LIC, ketoprofen (IS)	[65]
Carbamazepine	plasma or serum	One step extraction by simultaneous protein and phospholipids precipitation	UHPLC-MS/MS m/z: 237.2 → 194.15 (CBZ), 253.2 → 208.14 (OXC), 255.2 → 194.15 (MHD), 297.3→ 194.09 (ESL)	Intersil RP-HPLC (250 mm × 4.6 mm; 5 µm) acetonitrile-methanol-ammonium acetate in water (32:3:65, v/v/v)	0.5 mg/L; 0.5–40 mg/L	TDM	OXC, ESL, MHD, gatifloxacin (IS)	[52]
Carbamazepine	plasma and saliva	Protein precipitation with methanol	LC-MS/MS m/z: 237 → 194 (CBZ), 253 → 180 (CBZE), 251 → 108 (LCM)	Zorbax SB-C18 (100 mm × 3 mm, 3.5 µm) 0.1% formic acid in water and methanol (35:65, v/v)	1.1 mg/L; 1.1–17.6 mg/L	Preclinical pharmacokinetic studies and TDM	CBZE, LCM (IS)	[53]

Table 2. *Cont.*

Carbamazepine	plasma	LLE with ethyl acetate	SFC-ESI-MS/MS (supercritical fluid chromatography/mass spectrometry m/z: 237.2 → 194.1 (CBZ), 253.1 → 180.1 (OXC), 255.1 → 193.0 (MHD) 338.2 → 78.0 (TPM) 285.2 → 193.1 (IS)	UPC2TM BEH, 2EP (100 mm × 3 mm; 1.7 µm) carbon dioxide and methanol	0.01 mg/L; 0.01–15 mg/L	TDM	TPM, OXC, MHD, diazepam (IS)	[54]
Carbamazepine	DBS	LLE with methanol and ammonium formate with 0.15% formic acid in water using Whatman TM903 protein saver card	LC-MS/MS m/z: 237.2 → 178.9 (CBZ–I), 237.3 → 194.1 (CBZ–III), 245.2 → 200.1 (CBZ-d8) 247.4 → 204.1 (CBZ-d10), LTG-13C_3 (IS), LEV-d6 (IS), VPA-d6 (IS)	Acquity UPLC BEH, C18 (50 mm × 2.1 mm, 1.7 µm) 10 mM ammonium formate with 0.15% formic acid and 100% methanol	2.5 µM/L (0.6 mg/L); 5–30 µM/L (1.2-7.1 mg/L)	Routine laboratory DBS analysis as an alternative matrix	LTG, LEV and VPA CBZ-d8 (IS), CBZ-d10 (IS), LTG-13C_3 (IS), LEV-d6 (IS), VPA-d6 (IS)	[64]
Carbamazepine	exhaled breath condensate (EBC)	Samples directly analyzed without pretreatment	HPLC-FLD $\lambda_{ex}/\lambda_{em}$ = 290/480 nm	Copper nanoclasters (CuNCs) coated with cetyl trimethylammonium bromide with 0.1 M/L phosphate buffer (pH = 5)	0.08 mg/L; 0.2-20 mg/L	Routine quantification of CBZ in clinical practices using a non-invasive sampling method	None	[61]
Cenobamate	plasma (rats)	Protein precipitation with acetonitrile	LC-MS/MS m/z: 268.06 → 198 (CNB), 216.09 → 198.01 (IS)	Spursil C-18 10 mM ammonium formate and acetonitrile (60:40, v/v)	NA; 0.01-5 mg/L	Pharmacokinetic studies in healthy subjects	carisbamate (IS)	[186]
Cenobamate	plasma	Protein precipitation	LC-MS/MS	NA	0.02 mg/L; 0.02-10 mg/L	Pharmacokinetic studies in healthy subjects	GBP, LEV, PGB	[2]
Cenobamate	whole blood, plasma, urine and faeces	Protein precipitation	LC-MS/MS coupled with radio flow-through detector	NA	NA; 0.08-40 mg/L	Pharmacokinetics and mass balance assessment in healthy male subjects	CNB's eight metabolites	[187]
Clobazam	serum	LLE with ethyl acetate and reconstitution with acetonitrile:water (80:20, v/v)	LC-MS/MS m/z: 301 → 259 (CLB), 287 → 245 (N-CLB)	Symmetry C18 (75 mm × 4.6 mm, 3.5 µm) methanol:water:acetonitrile (50:30:20, v/v/v) with 0.05% formic acid	0.025 mg/L; 0.025–0.525 mg/L	TDM, applied in the clinical laboratory routine	Clonazepam, N-CLB, tenazepam (IS), clonazepam-d4 (IS), clonazepam-8-chloroisomer-13C_6 (IS)	[84]

Table 2. *Cont.*

Clobazam	plasma	Protein precipitation with methanol	LC-MS/MS m/z: 301 → 259 (CLB), 287 → 245 (N-CLB)	Phenomenex Kinetex Biphenyl (50 mm × 2.1 mm, 1.7 μm) 5 mM ammonium formate with 0.01% ammonium hydroxide and methanol	0.002 mg/L; 0.002–0.75 mg/L	Bioequivalence/pharmacokinetic studies	N-CLB	[85]
Eslicarbazepine acetate	plasma	SALLE	HPTLC-UV λ=217 nm (ESL and OXC) λ= 265 nm (CBZ and OXC)	Pre-coated silica gel plate G 60-F_{254} (20 cm x20 cm, 6–8 μm) n-hexane- methylene chloride—ethanol-glacial acetic acid (50:40:10:0.1 v/v/v/v)	72.82 ng/spot (14.56 mg/L); 150–1000 ng/spot (30–200 mg/L)	Clinical study in epileptic patients and pharmaceutical sample	OXC, CBZ	[49]
Eslicarbazepine acetate	plasma or serum	One step extraction by simultaneous protein and phospholipids precipitation	UHPLC-MS/MS m/z: 237.2 → 194.15 (CBZ), 253.2 → 208.14 (OXC), 255.2 → 194.15 (MHD), 297.3→ 194.09 (ESL)	Intersil RP-HPLC (250 mm × 4.6 mm; 5 μm) acetonitrile-methanol-ammonium acetate in water (32:3:65, v/v/v)	0.5 mg/L; 0.5–40 mg/L	TDM	CBZ, OXC, MHD, gatifloxacin (IS)	[52]
Eslicarbazepine acetate	postmortem blood, serum and plasma	Protein precipitation with methanol	LC-MS/MS m/z: 237.3 → 194.2 (CBZ), 253.1 → 236.1 (CBZE), 297.2 → 194.1 (ESL), 172.2 → 154.2 (GBP), 251.1 → 108.1 (LCM), 171.1 → 154.0 (LEV), 256.1 → 166.0 (LTG), 253.2 → 180.1 (OXC), 231.1 → 188.2 (PB), 160.2 → 142.2 (PGB), 217.2 → 159.2 (STP), 376.1 → 247.1 (TGB), 143.1 → 143.1 (VPA), 130.1 → 71.2 (VGB), 211.2 → 119.1 (ZNS)	Phenomenex Gemini C18 (150 mm × 2.1 mm, 5 μm) 2 mM ammonium acetate in water/2 mM ammonium acetate in methanol), gradient mode	0.5 mg/L; 0.5–50 mg/L	Routine forensic toxicology and therapeutic drug monitoring	CBZ, CBZE, ESL, OXC, S-licarbazepine, GBP, LCM, LTG, LEV, PGB, PB, PHT and its metabolite, retigabine and metabolite, STP, TPM, TGB, VPA, VGB, ZNS, tolbutamide (IS), 10,11-dihydrocarbamazepine, GBP-d10	[81]

Table 2. *Cont.*

Ethosuximide	plasma	Microextraction with toluene combined with derivatization	LC-MALDI-TOF MS for analysis of ESM in plasma (MRM 190.05 and 379.09) and nano UPLC-LTQ Orbitrap for protein modification analysis	Concentrated column Symmetry C18 (180 mm × 20 mm, 5 μm) Nano-flow column BEH (Ethylene Bridged Hybrid) C18 column (150 mm × 75 mm, 1.7 μm) 0.1% formic acid and acetonitrile with 0.1% formic acid	5 mg/L; 5–500 mg/L	Healthy volunteer after intake 500 mg ESM	ESM-d3 (IS)	[40]
Ethosuximide	plasma dried plasma spots (DPS)	Methanol protein precipitation	LC-UV λ=210 nm	Column XBrigde C18 (250 mm × 4.6 mm, 3.5 μm) acetonitrile and 50 mM phosphate buffer at pH = 4.5	9.6 mg/L; 9.6–192 mg/L	TDM—epilepsy patients undergoing mono- or polytherapy	Linezolid (IS), LEV, LTG, FBM, RFM, ZNS and monohydroxycarbamaz epine	[41]
Felbamate	plasma	Protein precipitation with acetonitrile	HPLC-UV λ=210 nm	Synergi Hydro-RP column (150 mm × 4.0 mm, 4 μm) potassium dihydrogen phosphate buffer 50 mM pH = 4,5/acetonitrile/metha nol (65:35, v/v)	5 mg/L; 30–80 mg/L	Therapeutic drug monitoring in plasma of 655 patients with epilepsy	LTG, 10,11-dihydro-10-hydroxy-5H-dibenzo[b,f]azepine-5-carboxamide, OXC metabolite), 4-methylprimidone (IS)	[123]
Felbamate	plasma and DPS	Protein precipitation with methanol	HPLC-UV λ=210 nm	XBridge C18 (250 mm × 4.6 mm, 3.5 μm) 50 mM phosphate buffer at Ph = 4.5/acetonitrile, gradient mode	9.6 mg/L (plasma and DPS); 2.4–96 mg/L (plasma and DPS)	TDM	LEV, LTG, ESM, RFM, ZNS, CBZ, linezolid (IS)	[41]

Table 2. *Cont.*

Felbamate	plasma	Protein precipitation with methanol	UHPLC-MS/MS m/z: 239.2 → 178.2 (FBM), 237.1 → 194.2 (CBZ), 253.2 → 180.2 (CBZE), 301.2 → 259.2 (CLB), 142.0 → 72.0 (ESM), 172.2 → 137.2 (GBP), 256.1 → 211.1 (LTG), 171.1 → 126.1 (LEV), 231,2 → 188.1 (PB), 253.2 → 182.2 (PHT), 219.2 → 162.2 (PRM), 376.2 → 149.1 (TGB), 340.2 → 264.2 (TPM), 143.1 → 143.1 (VPA), 130.1 → 71.2 (VGB), 213.1 → 132.1 (ZNS)	Acquity UPLC BEH C18 (50 mm × 2.1 mm, 1.7 µm) 10 mM ammonium acetate with 0,1% formic acid/methanol, gradient mode	4.2 mg/L; 4.2–105 mg/L	TDM	CBZ, CBZE, CLB, clonazepam, diazepam, ESM, GBP, LTG, LEV, nitrazepam, PB, PHT, PRM, TGB, TPM, VPA, VBG, ZNS	[67]
Gabapentin	serum	Protein precipitation with acetonitrile	UHPLC-MS/MS m/z: 172 → 154 (GBP), 182 → 147 (GBP-d10), 256 → 43 (LTG), 261 → 48 (LTG-13C,15N$_4$), 171 → 69 (LEV), 177 → 69 (LEV-d6), 213 → 132 (ZNS), 219 → 138 (ZNS-13C6)	ACQUITY UPLC BEH C18 column (30 mm × 2.1 mm, 1.7 µm) 2 mM ammonium acetate in water and 2 mM ammonium acetate in methanol, both containing 0.1% formic acid, gradient elution	0.1 mg/L; 0.1–100 mg/L	10 epileptic patients	LTG, LEV, ZNS and monohydroxy derivative of OXC	[75]
Gabapentin	blood	Protein precipitation with methanol	LC-MS/MS m/z: 172.1 → 154.1 (GBP), 160.1 → 142.1 (PGB), 182.2 → 164.2 (GBP-d10), 166.2 → 148.1 (PGB-d6) LC-TOF-MS	Poroshell 120 EC C-18 (100 mm × 2.1 mm, 2.7 µm) water and acetonitrile containing 0.1% formic acid	0.5 mg/L; 0.5–50 mg/L	1091 blood samples for toxicological analysis	GBP-d10 (IS), PGB, PGB-d6 (IS)	[139]
Gabapentin	blood	Protein precipitation with acetonitrile	LC-MS/MS m/z: 172.1 → 154.0 (GBP), 160.1 → 55.2 (PGB), 182.1 → 164.0 (GBP-d10), 166.2 → 148.1 (PGB-d6)	Poroshell 120 EC C-18 (100 mm × 2.1 mm, 2.7 µm) water and acetonitrile containing 0.1% formic acid	0.5 mg/L; 0.5–50 mg/L	Blood samples for toxicological analysis	Baclofen, GBP-d10 (IS), PGB, PGB-d6 (IS)	[140]
Gabapentin	serum	LLE with hexanol followed by derivatization with hexyl chloroformate	GC-MS m/z: 240 (GBP), 228 (PGB), 212 (VGB), 184 (IS)	HP5-MS (30 m × 0.25 mm, 0.25 µm) Carrier gas: helium	0.5 mg/L; 0.5–50 mg/L	Therapeutic drug or compliance monitoring	PGB, VGB, 3-(4-chlorophenyl)-propionic acid (IS)	[141]
Gabapentin	DBS	microwave-assisted derivatization with heptafluorobutanol	GC-MS m/z: 195 (GBP), 205 (GBP-d10)	HP5-MS (30 m × 0.25 mm, 0.25 µm) Carrier gas: helium	1 mg/L; 1–30 mg/L	15 healthy volunteers	GBP-d10 (IS)	[142]

Table 2. *Cont.*

Lacosamide	postmortem whole blood, clinical serum and plasma	LLE with alkaline condition (0.01 M NaOH pH 12) and ethyl acetate	GC-MS m/z: 91 (LCM), 100 (IS)	HP-5 MS UI (30 m × 0.25 mm, 0.25 µm) Carrier gas: helium	0.5 mg/L; 2–100 mg/L	Routine forensic toxicology and therapeutic drug monitoring	Moclobemide (IS)	[165]
Lacosamide	plasma	SPE through HF Bond Elut C18 and derivatization using N-methyl-N-tert-butyldimethylsilyltrifluoroacetamide with 1% tert-butyldimethylsilylchloride in acetonitrile	GC-MS m/z: 91 (LCM), 132 (IS)	DB-5 MS (30 m × 0.25 mm, 0.25 µm) Carrier gas: helium	0.2 mg/L; 0.2–20 mg/L	Therapeutic drug monitoring	LEV-d6 (IS)	[167]
Lacosamide	serum	Protein precipitation with methanol and SPE-mass spectrometry online	LC-MS/MS m/z: 251.1 → 108.1 (LCM), 255.1 → 91.0 (IS)	Phenyl SPE cartridge with water containing 10 mM ammonium acetate, 0.1% formic acid and 0.01% trifluoroacetic acid	0.05 mg/L; 5–50 mg/L	Therapeutic drug monitoring	Lacosamide-13C, d3 (IS)	[166]
Lacosamide	plasma	Protein precipitation with methanol and LLE with ethyl acetate	HPLC-DAD λ=220 nm (LCM, LEV) λ=239 nm (ZNS, IS)	LiCHroCART Purospher Star C18 (55 mm × 4 mm, 3 µm) water/acetonitrile, gradient mode	0.5 mg/L; 5–300 mg/L	Pharmacokinetic studies in human and for therapeutic drug monitoring	LEV, ZNS, antipyrine (IS)	[105]
Lacosamide	urine and bulk	Protein precipitation with methanol	UHPLC-DAD λ=205 nm	Hypersil BDS C18 (150 mm × 4.6 mm, 5 µm) 0.05 M/L phosphate buffer pH = 6,50/methanol/acetonitrile (80:10:10, v/v/v)	0.093 mg/L; 0.1–70 mg/L	Application to pharmaceutical dosage form (tablets) and human urine LCM determination	LEV, catechol (IS)	[103]
Lamotrigine	plasma	Deproteinization with 10% acetic acid followed by LLE with diethyl ether:dichloromethane (64:36)	HPLC-UV λ=210 nm	RP-18 column (250 mm) acetonitrile and 0.1M potassium dihydrogen phosphate (25:75, v/v)	2 mg/L; 2–50 mg/L	22 epileptic patients	Barbital sodium (IS)	[151]
Lamotrigine	plasma	Protein precipitation using methanol with 1% acetic acid	HPLC-UV λ=210 nm	Acclaim C-18 (150 mm × 4.6 mm, 5 µm) potassium dihydrogen phosphate buffer (50 mM) and methanol (61:39, v/v)	NA; 2.4–120 mg/L	186 clinical samples	OXC, 10,11-dihydro-10-hydroxycarbazepine, Fluconazole (IS)	[71]

Table 2. *Cont.*

Lamotrigine	plasma	Protein precipitation with methanol	HPLC-UV λ=210 nm	XBridge C18 (250 mm × 4.6 mm, 3.5 μm acetonitrile and 50 mM/L phosphate buffer at pH = 4.5, gradient elution	0.6 mg/L; 0.6–24 mg/L	61 epileptic patients	Linezolid (IS)	[152]
Lamotrigine	plasma	Protein precipitation with methanol	HPLC-UV λ=260 nm	Diamonsil C_{18}(150 mm × 4.6mm, 5 μm) 0.1% trifluoroacetate and methanol (59:41, v/v)	1 mg/L; 1–50 mg/L	67 patients with epilepsy	Diazepam (IS)	[153]
Lamotrigine	serum	LLE with diethyl ether	HPLC-UV λ=220 nm	Capcell Pak C18 (250 mm × 4.6 mm, 5 μm) acetonitrile and 0.05 M NaH_2PO_4 (26.5:73.5, v/v, pH = 4.5)	NA	214 epileptic patients	Chloroxazone (IS)	[154]
Lamotrigine	plasma	Protein precipitation with acetonitrile	LC-MS/MS m/z: 256.1 → 58.0 (LTG), 237.1 → 194.1 (CBZ), 247.2 → 204.1 (CBZ-d10)	XSelect CSH C18 XP (100 mm × 2.1 mm, 2.5 μm) 5 mM ammonium acetate (with 0.1% of formic acid) and acetonitrile, gradient elution	0.005 mg/L; 0.005–10.5 mg/L	TDM in schizophrenic patients	CBZ, CBZ-d10 (IS), antipsychotics, antidepressants, anxiolytics	[156]
Levetiracetam	plasma	Protein precipitation with acetonitrile	LC-MS/MS m/z: 171.1 → 154.1 (LEV), 172.5 → 126.1 (UCB L057), 256.3 → 167.3 (IS)	Agilent Zorbax SB-C18, (100 mm × 2.1 mm, 3.5 μm) 0.1% formic acid in water with acetonitrile (40:60, v/v), isocratic elution	0.5 mg/L; 0.5–100 mg/L	Pharmacokinetic study of LEV in patients with epilepsy	LEV metabolite (UCB L057), Diphenhydramine (IS)	[100]
Levetiracetam	plasma	Protein precipitation and LLE with methanol and ethyl acetate	HPLC-DAD λ=220 nm (LCM, LEV) λ=239 nm (ZNS, IS)	LiChroCART®Purospher®StarC18 (55 mm × 4 mm, 3 μm) water/acetonitrile (90:10, v/v), gradient elution	2.5 mg/L; 2.5–40 mg/L	Therapeutic drug monitoring in 11 distinct epileptic patients	Antipyrine (IS), LCM, ZNS	[105]

Table 2. *Cont.*

Levetiracetam	plasma/serum	Protein precipitation with methanol	HPLC-UV λ=205 nm	Venusil XBP C18, (250 mm × 4.6 mm, 5 µm) 50 mM pH = 5.5 potassium dihydrogen phosphate-acetonitrile (90:10, v/v), isocratic elution	1 mg/L; 1–60 mg/L	Therapeutic drug monitoring in epilepsy patients	Gabapentin (IS)	[108]
Levetiracetam	plasma	Protein precipitation with zinc sulphate	HPLC-UV λ (NA)	LichroCART 250–4.6 RP-18 (250 mm × 4.6 mm, 5 µm) 50 mM pH = 4.5 potassium dihydrogen phosphate buffer and acetonitrile/methanol (3/1) (65:35, v/v), isocratic elution	5 mg/L; 5–80 mg/L	Therapeutic drug monitoring in epilepsy patients in daily clinical practice	NA	[109]
Levetiracetam	plasma and CRRT effluent sample	Protein precipitation with acetonitrile	HPLC-UV λ=210 nm	Synergi Hydro-RP (150 mm × 4.6 mm, 4 µm) 50 mM phosphate buffer and acetonitrile, gradient elution	2 mg/L; 2–80 mg/L	Therapeutic drug monitoring in patients undergoing continuous renal replacement therapy (CRRT)	Caffeine (IS)	[110]
Oxcarbazepine	plasma	SALLE	HPTLC-UV λ=217 nm (ESL and OXC) λ= 265 nm (CBZ and OXC)	Pre-coated silica gel plate G 60-F_{254} (20 cm × 20 cm, 6–8 µm) n-hexane-methylene chloride-ethanol-glacial acetic acid (50:40:10:0.1, v/v/v/v)	34.58 ng/spot (6.92 mg/L); 85–1000 ng/spot (17–200 mg/L)	Clinical study in epileptic patients and pharmaceutical sample	ESL or CBZ mixture	[49]
Oxcarbazepine	plasma	Protein precipitation by methanol with 1% acetic acid	HPLC-UV λ=210 nm	Acclain C18 (Thermo, 150 mm × 4.6mm, 5 µm) potassium dihydrogen phosphate buffer (50 mM) and methanol (61:39)	2.4 mg/L; 2.4–120 mg/L	Clinical practice	LTG, MHD, fluconazole (IS)	[71]

Table 2. *Cont.*

Oxcarbazepine	plasma	MEPS	HPLC-DAD<br>λ=215, 237, 280 nm	LiChroCART Purosher Star RP acetonitrile (6%)/water-methanol-triethyloamine (94%) (73.2:26.5:0.3; v/v/v)	0.1 mg/L;<br>0.1–5 mg/L;	Routine TDM of CBZ with, LTG, OXC, PB and PHT	CBZ, LTG, PB, PHT, CBZE, licarbazepine, ketoprofen (IS)	[65]
Oxcarbazepine	plasma	Protein precipitation with acetonitrile	LC-MS/MS<br>m/z: 253.1 → 180.2 (OXC), 255.1 → 194.2 (HOXC)	Synergi Hydro-RP (50 mm × 2.0 mm, 4 μm) water-formic acid (100/0.1, v/v) and acetonitrile – methanol-formic acid (50/50/0.1, v/v/v)	0.02 mg/L;<br>0.02–10 mg/L	Clinical study, pharmacokinetic sample assay in order to support a clinical trial	HOXC	[66]
Oxcarbazepine	plasma	Protein precipitation with methanol	UHPLC-MS/MS<br>m/z: 253.1 → 179.9 (OXC), 255.9 → 210.8 (LTG); 254.8 → 193.7 (MHD); 357 → 263.7 (TPM); 170.9 → 125.8 (LVT); 173.9 → 128.8 (LVT-d3); 369 → 269.9 (TPM-d12) 217.9 → 185.7 (IS)	Waters BEH C18 (50 mm × 2.1 mm; 1.7 μm) water with 0.1% formic acid/methanol	0.20 mg/L;<br>0.20–20 mg/L	Clinical application in 259 samples from patients treated for epilepsy<br>TDM	LTG, LVT, TPM, MHD, LVT-d3 (IS), TPM-d12 (IS), 3,5-diamino-6-[2methoxyphenyl]-1,2,4-triazine (IS)	[74]
Oxcarbazepine	plasma	LLE with ethyl acetate	SFC-ESI-MS/MS (supercritical fluid chromatography/mass spectrometry<br>m/z: 253.1 → 180.1 (OXC) 237.2 → 194.1 (CBZ), 255.1 → 193.0 (MHD), 338.2 → 78.0 (TPM), 285.2 → 193.1 (IS)	UPC2TM BEH, 2EP (100 mm × 3 mm; 1.7 μm) carbon dioxide and methanol	0.01 mg/L;<br>0.01–8 mg/L	TDM, simultaneous quantification of several AED, pharmacokinetics study	CBZ, TPM, MHD, diazepam (IS)	[54]
Piracetam	plasma	Protein precipitation with 20% perchloric acid	HPLC-UV<br>λ = 200 nm	RP-18 Merck LiChroSpher 100 (250 mm × 4mm, 5 μm), gradient mode aqueous solution of 0,01% perchloric acid/methanol/aceton itrile	2 mg/L;<br>1–100 mg/L	Bioequivalence study	None	[88]
Piracetam	plasma and cerebrospinal fluid	Direct sample injection	Micellar electrokinetic chromatography (MEKC) Beckman P/ACE MDQ system with UV detection	Fused capillary (40.2 cm × 50 μm)	1 mg/L;<br>5–500 mg/L	Application to analyze piracetam in patients with aphasia	Imidazole (IS)	[89]

Table 2. *Cont.*

Piracetam	serum and urine (tablets, syrup)	Protein precipitation serum with acetonitrile, urine diluted	HPLC-UV λ=205 nm	Hibar Bondapak ODS C18 (250 mm × 4.6 mm, 5 µm) trimethylamine in water/acetonitrile (70:30, v/v) with phosphoric acid pH = 6,5	0.0093 mg/L; 0.02-10 mg/L	Application to analyze piracetam in human serum and urine (also in bulk drugs)	LEV	[90]
Perampanel	plasma	Deproteinization by acetonitrile	HPLC-FLD $\lambda_{ex}/\lambda_{em}$ = 290/430 nm	Phenomenex (100 mm × 4.6 mm, 2.6 µm) sodium acetate buffer–acetonitrile (40:60, v/v)	0.002 mg/L; 0.002-1 mg/L	Clinical study in 30 patients treated with PER (2–10 mg/d) receiving different AED co-therapy	Mirtazapine (IS)	[172]
Perampanel	plasma	LLE with diethyl ether	HPLC–FLD $\lambda_{ex}/\lambda_{em}$ = 290/430 nm	YMC pack pro C18 (150 mm × 4.6 mm, 5 µm) acetonitrile water-acetic acid-sodium acetate (840:560:3:1.8, v/v/v/w)	0.001 mg/L; 0.001-0.5 mg/L	Clinical studies and TDM at laboratories where LC-MS/MS system are not available	ER-167615 (IS)	[170]
Perampanel	DPS	LLE with methanol	HPLC-UV λ=320 nm	Reverse-phase monolithic column water-acetonitrile (60:40, v/v) with phosphoric acid	0.025 mg/L; 0.025-1 mg/L	TDM	None	[175]
Perampanel	serum	Acetonitrile stacking for on-line sample pre-concentration	CE-FL (Capillary electrophoretic methods with fluorescence detection) $\lambda_{ex}/\lambda_{em}$ = 240-400/495 nm	Fused–silica capillary Electolyte–50 mM chloroacetic acid with 0.5% polyvinylalcohol (pH = 2.15)	0.009 mg/L; 0.01–1 mg/L	TDM and toxicological analysis	None	[174]
Phenobarbital	whole blood	Fully automated dried blood spot extraction system. Volumetric absorption microsampling (VAMS), extraction with acetonitrile/water (80:20, v/v) with 5 mM ammonium acetate	UHPLC-MS/MS m/z: 231.0 → 187.8 (PHB), 143 → 143 (VPA), 251.1 → 102.2 (PHT), 237 → 194.1 (CBZ), 253.1 → 180 (CBZE), 148.9→ 148.9 (VPA-d6), 236 → 193 (PHB-d5), 261 → 218.1 (PHT-d10), 247 → 204.1 (CBZ-d10), 263.1 → 220.2 (CBZE-d10)	Chromolith reversed phase (RP-18) endcaped (100 mm × 4.6 mm) 5 mM ammonium acetate and 5 mM ammonium acetate in acetonitrile/water (95:5, v/v)	1.0 mg/L; 1.0–160.0 mg/L	Assessment levels of AEDs in patients	PHB-d5 (IS), CBZ, CBZ-d10, CBZE, CBZE-d10 VPA, VPA-d6, PHT, PHT-d10	[21,22]

Table 2. *Cont.*

Phenobarbital	plasma	SPE reversed-phase cartridges (Waters Oasis HLB) washed out by acetonitrile (10 mM ammonium buffer pH 3.5, 2:98 v/v)	online-SPE-LC-HRMS/MS m/z: 231.08 → 188.08 (PHB), 251.08 → 208.08 (PHT), 237.10 → 194.09 (CBZ), 253.09 → 210.09 (CBZE)	Zorbax SB-C-18 (250 mm × 4.6 mm, 5 μm) acetonitrile, methanol and 10 mM acetate buffer pH = 5.5 (10:20:70, v/v/v)	PHB, PHT 0.008 mg/L; 0.008–2.5 mg/L	TDM for epilepsy patients	PHT, CBZ, CBZE, LTG (IS)	[23]
Phenobarbital	plasma	Protein precipitation with acetonitrile	LC-MS/MS m/z: 231.1 → 231.1 (PHB), 253.1 → 182.1 (PHT), 237.2 → 194.1 (CBZ), 253.0 → 210.0 (CBZE), 256.1 → 43 (LTG), 253.1 → 208.0 (OXC), 255.1 → 194.1 (MHD), 171.1 → 126.1 (LEV), 143.1 → 143.1 (VPA), 338.2 → 78 (TPM)	Zorbax SB-C18 (50 mm × 4.6 mm, 2.7 μm) acetonitrile/water	0.15 mg/L; 0.4–60 mg/L	TDM for epilepsy patients	CBZ and its CBZE, LTG, OXC and 10-hydroxycarbazepine, LEV, PHT, VPA, TPM and diphenhydramine (IS)	[24]
Phenytoin	plasma	Protein precipitation with acetonitrile	LC-ESI-MS/MS m/z: 251.1 → 208.1 (PHT), 237.1 → 194.0 (CBZ), 255.1 → 237 (CBZ-OH), 256 → 211(LTG), 253.1 → 180.1(OXC), 171.1 → 126 (LEV), 143.1 → 143.1 (VPA), 338.1 → 78.1 (TPM), 211 → 119 (ZNS)	Phenomenex Kinetic C18 (100 mm × 2.1 mm, 2.6 μm) 5 mM ammonium and acetonitrile with 5 mM ammonium acetate	PHT, ZNS TPM: 0.01–10 mg/L VPA 0.05–50 mg/L LEV, LTG, CBZ, CBZE OXC: 5–5000 μg/L	Therapeutic drug monitoring in patients with epilepsy	ZNS, TPM, VPA, LEV, LTG, CBZ, CBZ-OH, OXC	[30]
Phenytoin	whole blood, plasma	DBS extracted of methanol/water (80:20, v/v) and 0.1% formic acid plasma diluted and precipitated methanol/water 0.1% formic acid	LC-MS/MS m/z: 253.2 → 182.2	Synergii Fusion column (50 mm × 2 mm, 4 μm) water/0.1% formic acid and methanol/0.1% formic acid	1.0 mg/L; 0.1–100 mg/L	Determination of PHT in paediatric patients	None	[31]
Phenytoin and phosphenytoin	plasma	free fraction of phenytoin obtained after ultrafiltration using Milipore protein filter	LC-MS/MS m/z: 252.98 → 182.1 (PHT), m/z: 253.98 → 104.0 (FOS)	Restek Ultra BiPh (50 mm × 2.1 mm, 5 μm) formic acid in water and formic acid in methanol	NA; 0.1–4 mg/L	Measurement of free phenytoin in human plasma	FOS	[32]
Pregabalin	plasma	Derivatization with 4-fluoro-7-nitrobenzofurazan	HPLC-FLD $\lambda_{ex}/\lambda_{em}$ = 470/530 nm	TSK-gel ODS-140HTP (50 mm × 2.1 mm, 2.3 μm) acetonitrile, methanol, and 50 mM/L phosphate buffer pH = 2	0.05 mg/L; 0.05–10 mg/L	40 patients with pain	Gabapentin (IS)	[143]

Table 2. *Cont.*

Pregabalin	plasma, DPS, DBS	plasma: oasis mixed-mode cation exchange (MCX) extraction cartridge bed<br>dry blood/plasma spots: LLE with methyl tert-butyl ether and diethyl ether (80/20, v/v)	HPLC-MS/MS<br>m/z: 160.1 → 141.9 (PGB), 164.1 → 145.9 (IS)	Poroshell 120 EC-C18 methanol, acetonitrile, and 5 mM ammonium formate solution (80/10/10, v/v/v)	Plasma:<br>0.02–16 mg/L<br>Blood/plasma spots:<br>0.01–10 mg/L	Bioequivalence study performed in 14 healthy human volunteers after administration of 300 mg PGB	Pregabalin-d4 (IS)	[144]
Pregabalin	DPS, DBS	Derivatization with n-propyl chloroformate in the presence of n-propanol followed by LLE with ethyl acetate	HPLC-MS/MS<br>m/z: 288.00 → 228.04 (derivatized PGB), 272.03 → 212.00 (derivatized IS)	YMC-Pack Octyl column (50 mm × 4.0 mm, 3 µm) acetonitrile and 0.15% formic acid	DBS: 0.2 mg/L;<br>0.2–20 mg/L<br>DPS:<br>0.4 mg/L;<br>0.4–40 mg/L	12 epileptic patients	4-aminocyclohexanecarboxylic acid (IS)	[145]
Primidone	plasma	Online extraction with use restricted access carbon nanotubes (RACNTs)	LC-UV<br>λ=210 nm	C18 (250 × 4.6 mm, 5 µm) column in switching system monopotassium phosphate buffer 0.01 M/L, pH = 6.0: acetonitrile:methanol (55:25:20, v/v/v) and water or methanol:water (90:10, v/v).<br>30 mg of the RACNTs in a column (10 × 4.6 mm)	0.2 mg/L;<br>0.2–40 mg/L	TDM of PRM and PHB in patients with mental illness	Hydantoin (IS),<br>PHB, CBZ	[36]
Primidone	plasma and DPS	On dried sample spot devices (DSSDs) and plasma extracted with acetonitrile	LC-MS/MS<br>m/z: 219.0 → 119.0 (PRM), 256.0 → 210.9 (LTG), 253.2 → 180.0 (OXC), 171.1 → 154.0 (LEV), 140.0 → 140.0 (ESM), 338.0 → 280.0 (TPM), 210.99 → 147.1 (ZNS), 171.1 → 154.0 (LEV), 251.05 → 108.1 (LCM), 239.0 → 107.1 (RFM), 255.0 → 108.1 (10-OH-OXC)	C18 Hypersil Gold column (50 mm × 2.1 mm, 1.9 µm) water/0.1% formic acid and acetonitrile/0.1% formic acid	0.32 mg/L;<br>0.7–26.3 mg/L	TDM in 129 undergoing mono and polytherapy for epilepsy	LCM-d3 (IS), LCM, LEV, ESM, ZNS, RFM, LTG, 10-OH-OXC, OXC, TPM	[37]

Table 2. *Cont.*

Rufinamide	brain tissue and plasma from rats	Protein precipitation with methanol	HPLC-UV λ=215 nm	Phenomenex Kinetex C18 (250 mm × 4.6 mm, 5 µm) 10 mM ammonium acetate buffer (pH = 4.7 ± 0.1, adjusted with glacial acetic acid) and acetonitrile (84.7:15.3, v/v), isocratic elution	0.0138 mg/L (plasma); 105.24 ng/g (brain); 0.1–2 mg/L (plasma) 300–6000 ng/g (brain)	Pharmacokinetic studies	Piribedil (IS)	[181]
Rufinamide	mouse plasma and tissues (brain, liver, kidney)	Protein precipitation with acetonitrile and LLE (extracted dichloromethane)	HPLC-UV λ=210 nm	LiChroCART Purospher Star column C18 (55 mm × 4 mm, 3 µm) water/acetonitrile (82:18, v/v), isocratic elution	0.1 mg/L; 0.1–30 mg/L	Preliminary pharmacokinetic studies to support non-clinical pharmacokinetic based studies on RFM	Chloramphenicol (IS)	[178]
Rufinamide	human, rat and rabbit plasma	Protein precipitation with methanol	LC-MS/MS m/z: 239 → 127 (RFM), 251 → 108 (IS)	Zorbax SB-C18 (100 mm × 3 mm, 3.5 µm) water with 0,5% formic acid/methanol (50:50, v/v)	0.005 mg/L; 0.04–2 mg/L	Therapeutic drug monitoring	Lacosamide (IS)	[179]
Rufinamide	postmortem whole blood, clinical serum and plasma	Protein precipitation with methanol	LC-MS/MS m/z: 237.3 → 194.2 (CBZ), 253.1 → 236.1 (CBZE), 297.2 → 194.1 (ESL), 172.2 → 154.2 (GBP), 251.1 → 108.1 (LCM), 171.1 → 154.0 (LEV), 256.1 → 166.0 (LTG), 253.2 → 180.1 (OXC), 231.1 → 188.2 (PB), 160.2 → 142.2 (PGB), 217.2 → 159.2 (STP), 376.1 → 247.1 (TGB), 143.1 → 143.1 (VPA), 130.1 → 71.2 (VGB), 211.2 → 119.1 (ZNS)	Phenomenex Gemini C18 (150 mm × 2.1 mm, 5 µm) 2 mM ammonium acetate in water/2 mM ammonium acetate in methanol), gradient mode	0.5 mg/L; 0.5–50 mg/L	Routine forensic toxicology and therapeutic drug monitoring	CBZ, CBZE, ESL, OXC, S-licarbazepine, GBP, LCM, LTG, LEV, PGB, PB, PHT and its metabolite, retigabine and metabolite, STP, TPM, TGB, VPA, VGB, ZNS, tolbutamide (IS), 10,11-dihydrocarbamazepine, GBP-d10	[81]
Rufinamide	plasma and DPS	On dried sample spot devices (DSSDs) and plasma extracted with acetonitrile	LC-MS/MS m/z: 239.0 → 107.1 (RFM), 256.0 → 210.9 (LTG), 253.2 → 180.0 (OXC), 171.1 → 154.0 (LEV), 140.0 → 140.0 (ESM), 338.0 → 280.0 (TPM), 210.99 → 147.1 (ZNS), 171.1 → 154.0 (LEV), 251.05 → 108.1 (LCM), 219.0 → 119.0 (PRM), 255.0 → 108.1 (10-OH-OXC)	C18 Hypersil Gold column (50 mm × 2.1 mm, 1.9 µm) water/0.1% formic acid and acetonitrile/0.1% formic acid	0.65 mg/L; 1.3–42.4 mg/L	Therapeutic drug monitoring	LCM-d3 (IS), LCM, LEV, ESM, ZNS, PRM, LTG, 10-OH-OXC, OXC, TPM	[37]

Table 2. *Cont.*

Rufinamide	plasma and DPS	Methanol protein precipitation	HPLC-UV λ=210 nm	Column Xbridge C18 (250 mm × 4.6 mm, 3.5 µm) Acetonitrile and 50 mM phosphate buffer at pH = 4.5	9.6 mg/L; 9.6–192 mg/L	TDM–epilepsy patients undergoing mono- or polytherapy	Linezolid (IS), LEV, LTG, FBM, ZNS and CBZ-OH	[41]
Stiripentol	plasma	Protein precipitation with acetonitrile	HPLC-FLD $\lambda_{ex}/\lambda_{em}$ = 200/400 nm	Inertsil® ODS-80A (10 mm × 4.6 mm, 5 µm) 25 mM phosphate buffer (pH = 2.6) and acetonitrile (43:57, v/v)	0.05 mg/L; 0.05–40 mg/L	37 patients with Dravet syndrome	None	[184]
Sulthiame	whole blood, plasma, urine	Protein precipitation with methanol (plasma) hemolyzed whole blood, or hemolyzed red blood cells urine was vortexed, sonificated and after centrifugation diluted	LC-MS/MS m/z: 289.0 → 225.1	XSelect HSS T3 water/acetonitrile	0.01 mg/L; 0.1–50 mg/L	Oral doses of 50, 100, and 200 mg of sulthiame tablets were administered healthy adult male volunteers	sulthiame-d4 (IS)	[42]
Tiagabine	postmortem blood, serum and plasma	Protein precipitation with methanol	LC-MS/MS m/z: 237.3 → 194.2 (CBZ), 253.1 → 236.1 (CBZE), 297.2 → 194.1 (ESL), 172.2 → 154.2 (GBP), 251.1 → 108.1 (LCM), 171.1 → 154.0 (LEV), 256.1 → 166.0 (LTG), 253.2 → 180.1 (OXC), 231.1 → 188.2 (PB), 160.2 → 142.2 (PGB), 217.2 → 159.2 (STP), 376.1 → 247.1 (TGB), 143.1 → 143.1 (VPA), 130.1 → 71.2 (VGB), 211.2 → 119.1 (ZNS)	Phenomenex Gemini C18 (150 mm × 2.1 mm; 5 µm) 2 mM ammonium acetate in water/2 mM ammonium acetate in methanol	0.05 mg/L; 0.05–10.0 mg/L	Forensic and toxicological analysis, TDM	CBZ, CBZE, ESL, OXC, S- licarbazepine, GBP, LCM, LTG, LEV, PGB, PHB, PHT and its metabolite 5-(p-hydroxyphenyl)-5-phenylhydantoin, retigabine (ezogabine) and its metabolite N-acetyl retigabine, RFM, STP, TPM, VPA, VGB, ZNS	[81]
Topiramate	plasma	LLE using dichloromethane and derivatization with 4-chlor-7-nitrobenzo-furazan	LC- FLD $\lambda_{ex}/\lambda_{em}$ = 475/530 nm	Reversed phase column Eclipse18 (150 mm × 4.6 mm, 5 µm) 0.05 M potassium phosphate buffer, pH = 5.5 and acetonitrile (61.5/38.5, v/v)	0.01 mg/L; 0.01–24 mg/L	Therapeutic drug monitoring in 27 patients with epilepsy	bendroflumethiazide (IS)	[114]

Table 1. *Cont.*

Topiramate	plasma	LLE with ethyl acetate and diethylether (95:5, v/v)	UHPLC-MS/MS m/z: 338.1 → 77.9 (TPM), 298.1 → 77.9 (M1, M2), 354.1 → 77.9 (M3, M4), 350.1 → 77.9 (IS)	Kinetex C-18 (50 mm × 2.1 mm, 2.6 μm) water and methanol	TPM 0.1–20 mg/L TPM metabolites: 2,3-desisopropylidene TPM (M1) 0.01–2 mg/L 4,5-desisopropylidene TPM (M2), 10-OH TPM (M3) and 9-OH TPM (4) M2, M3, and M4 0.001–0.2 mg/L	10 samples from patients with epilepsy	TPM-d12 (IS) and TPM metabolites: M1, M2, M3 and M4	[115]
Topiramate	plasma, whole blood	LLE of 8mm DBS with ethyl acetate followed by flash methylation with TMAH (trimethylanilinium hydroxide solution)	GC-MS m/z: 171, 229, 352	DB-5 MS (30 m and 0.25 μm)	0.5 mg/L; 0.5–30 mg/L	Adult volunteer (Hct=44%) after a single oral dose of 100 mg	5-(p-methylphenyl)-5-phenyl-hydantoin (IS)	[116]
Topiramate	plasma	Protein precipitation with acetonitrile	LC-MS/MS m/z: 338.3 → 77.9 (TPM), 150.0 → 91.0 (phentermine), 452.1 → 344.3 (doxazosin), 355.0 → 41.9 (pioglitazone)	60–5CN (100 mm × 2.1 mm, 5 μm) acetonitrile/20 mM ammonium formate with 0.3% formic acid (40:60, v/v)	1 μg/L; NA	12 healthy male volunteers after single oral dose phentermine and TPM (7,5/46 mg) in extended release capsules	Phentermine, pioglitazone (IS) and doxazosin (IS)	[117]
Topiramate	plasma	Plasma samples buffered with a TRIS buffer at a pH 8.2 and LLE with methyl terc-butyl ether	Capillary electrophoresis with capacitively-coupled contactless conductivity detection (CE-C4D), separation voltage 20 kV	Background electrolyte (BGE) composed of 15 mM triethylamine pH = 11.3, hydrodynamic injections by pressure (0.8 psi for 5 s)	1.0 mg/L; 1–30 mg/L	Plasma samples from hospital patients under treatment with TPM	IS-2-naphtol, PHT, CBZ, LTG, PHB LEV, GBP, OXC, VPA, clonazepam, CLB, diazepam, fluoxetine, omeprazole, venlafaxine, folic acid, captopril and diclofenac	[118]
Valproic acid	plasma	Protein precipitation with trifluoroacetic acid (TCA) followed by liquid-liquid microextraction (LLME) with chloroform	GC-FID	HP-5 (30 m × 0.32 mm, 0.25 μm) Carrier gas: nitrogen	0.2 mg/L; 0.2–100 mg/L	70 epileptic patients (1–18 years)	3-heptanone (metabolite)	[127]
Valproic acid	plasma	Protein precipitation with acetonitrile followed by dispersive liquid-liquid microextraction (DLLME) with chloroform	GC-FID	HP-5 (30 m × 0.32 mm, 0.25 μm) Carrier gas: nitrogen	6 mg/L; 6 - 140 mg/L	One epileptic patient treated with 125 mg valproic acid	None	[128]

Table 2. *Cont.*

Valproic acid	plasma	LLE with chloroform	GC-FID	Gs-BP 100% dimethylpolysiloxane (10 m × 0.53 mm, 2.65 μm) Carrier gas: helium	5 mg/L; 5–320 mg/L	50 epileptic patients	Octanoic acid (IS)	[129]
Valproic acid	plasma	LLE using n-hexane	HPLC-UV λ=210 nm	C-8 Symmetry (150 mm × 3.9 mm, 5 μm) 40 mM sodium dihydrogen phosphate pH = 3.5 and acetonitrile (56:44, v/v)	2 mg/L; 2–200 mg/L	Pharmacokinetic study of one healthy subject after administration of 500mg extended release VPA	Nonanoic acid (IS)	[130]
Valproic acid	saliva, serum	Protein precipitation with acetonitrile	HPLC-UV λ=210 nm	Chromolith RP 18e (100 mm × 4,6 mm) acetonitrile and 0.05 M potassium dihydrogen ortho phosphate (pH = 3.0) (45:55, v/v)	NA; 5–100 mg/L	65 epileptic patients (9–62 years)	None	[131]
Valproic acid	plasma	SPE	HPLC-MS/MS m/z: 143.0 → 143.0 (VPA), 140.9 → 140.9 (2-ene-VPA, 4-ene-VPA), 283.9 → 239.9 (IS)	Poroshell SB-C18 column (50 mm × 4.6 mm, 2.7 μm) water-acetonitrile	NA; 20–125 mg/L	60 epileptic patients (mean age 30 years old) treated with valproic acid 500 mg twice daily	VPA metabolites: 2-ene and 4-ene VPA; Probenecid (IS)	[132]
Valproic acid	serum	SPE	UHPLC-MS/MS m/z: 143.0 → 143.0 (VPA), 141.0 → 141.0 (2-ene-VPA, 4-ene-VPA), 138.8 → 138.8 (2,4-diene-VPA), 136.6 → 92.5 (IS)	RHD EC-C18 column (75 mm × 2.1 mm, 1.8 μm) acetonitrile and 10 mM ammonium acetate	NA; 1–200 mg/L	170 epileptic patients	VPA metabolites: 4-ene-VPA, 2,4-diene-VPA, 2-ene-VPA; Salicylic acid (IS)	[133]
Vigabatrin	human plasma, rat plasma, brain and retina	Protein precipitation with acetonitrile followed by derivatization with naphthalene 2,3-dicarboxaldehyde	HPLC-FLD $\lambda_{ex}/\lambda_{em}$ = 400/500 nm	Zorbax Eclipse AAA (150 mm × 4.6 mm, 3.5 μm) methanol and 10 mM potassium phosphate dibasic pH = 6.5, gradient elution	0.0646 mg/L; 0.0646–6.458 mg/L	Animal study after VGB intraperitoneal administration	GABA, taurine, gabapentin (IS)	[148]
(-)-R and (+)-S Vigabatrin	plasma	Protein precipitation with methanol followed by derivatization with o-phthaldialdehyde and N-acetyl-l-cysteine	UHPLC-MS/MS m/z: 391.1 → 149.7 (VGB), 397.1 → 268.0 (IS)	Kinetex EVO C-18 (100 mm × 2.1 mm, 1.7 μm) 5mM ammonium acetate and methanol:acetonitrile (63:37, v/v)	NA; 0.2–50 mg/L	29 children with West syndrome	Deuterated vigabatrin	[149]

Table 2. *Cont.*

Zonisamide	plasma	Protein precipitation with acetonitrile and then MEPS	HPLC-UV λ=240 nm	Acclaim RP 120 C18 (150 mm × 4.6 mm; 5 μm) acetonitrile-water (35:65, v/v)	0.2 mg/L; 0.2–80 mg/L	TDM in clinical practice	None	[158]
Zonisamide	plasma	Protein precipitation with methanol and LLE with ethyl acetate	HPLC-DAD λ=220 nm (LCM, LEV) λ=239 nm (ZNS, IS)	LiChroCART®Purospher®StarC18 (55 mm × 4 mm, 3 μm) water/acetonitrile (90:10, v/v), gradient elution	0.5 mg/L; 5–300 mg/L	Pharmacokinetic studies in human and for TDM	LEV, LCM, antipyrine (IS)	[105]
Zonisamide	serum	Protein precipitation with methanol	UHPLC-MS/MS m/z: 172 → 154 (GBP), 182 → 147 (GBP-d10), 256 → 43 (LTG), 261 → 48 (LTG-13C,15N$_4$), 171 → 69 (LEV), 177 → 69 (LEV-d6), 213 → 132 (ZNS), 219 → 138 (ZNS-13C6)	ACQUITY UPLC BEH C18 column (30 mm × 2.1 mm, 1.7 μm) 2 mM ammonium acetate in water and 2 mM ammonium acetate in methanol, both containing 0.1% formic acid, gradient elution	0.1 mg/L; 0.1–100 mg/L	10 epileptic patients	LTG, LEV, ZNS and MHD of OXC	[75]
Zonisamide	plasma	Reaction with 4-chloro-7-nitrobenzofurazan (NBD-Cl) chemosensor (0.2 M, pH 8 borate buffer with 0.2% methanolic NBD-Cl solution heated at 70°C in a water bath for 20 min, after cooling in ice bath, 0.2 M HCl, extracted by mixture of acetonitrile and methanol (1:1)	HPLC-FLD $\lambda_{ex}/\lambda_{em}$ = 465/550 nm	Zorbax RP-C18 (100 mm × 4.6 mm, 3.5 μm) acetonitrile:methanol :water (pH adjusted to 5 with 0.2 M phosphoric acid) (30:20:50% v/v)	0.086 mg/L; 0.1–3 mg/L	Therapeutic drug monitoring	TPM, sulpiride (IS)	[160]

References

1. XCOPRI. Full Prescribing Information. Available online: https://www.accessdata.fda.gov/drugsatfda_docs/label/2019/212839s000lbl.pdf (accessed on 13 July 2020).
2. Cenobamate (XCOPRI). Clinical Pharmacology and Biopharmaceutics Review(s); Application Number 212839Orig1s000. Available online: https://www.accessdata.fda.gov/drugsatfda_docs/nda/2019/ 212839Orig1s000ClinPharmR.pdf (accessed on 13 July 2020).
3. Cusumano, J.A.; Klinker, K.P.; Huttner, A.; Luther, M.K.; Roberts, J.A.; LaPlante, K.L. Towards precision medicine: Therapeutic drug monitoring–guided dosing of vancomycin and β-lactam antibiotics to maximize effectiveness and minimize toxicity. *Am. J. Health Syst. Pharm.* **2020**, *77*, 1104–1112, doi:10.1093/ajhp/zxaa128.
4. Reimers, A.; Berg, J.A.; Larsen Burns, M.; Brodtkorb, E.; Johannessen, S.I.; Johannessen Landmark, C. Reference ranges for antiepileptic drugs revisited: A practical approach to establish national guidelines. *DDDT* **2018**, *12*, 271–280, doi:10.2147/DDDT.S154388.

5. Johannessen Landmark, C.; Johannessen, S.I.; Patsalos, P.N. Therapeutic drug monitoring of antiepileptic drugs: Current status and future prospects. *Expert Opin. Drug Metab. Toxicol.* **2020**, *16*, 227–238, doi:10.1080/17425255.2020.1724956.
6. Serragui, S.; Lachhab, Z.; Soussi Tanani, D.; Cherrah, Y. Therapeutic Drug Monitoring of Antiepileptic Drugs: Indications and Modalities. *J. Pharm. Pharmacol. Res.* **2019**, *3*, 41–50, doi:10.26502/jppr.0019.
7. Knezevic, C.E.; Marzinke, M.A. Clinical Use and Monitoring of Antiepileptic Drugs. *J. Appl. Lab. Med.* **2018**, *3*, 115–127, doi:10.1373/jalm.2017.023689.
8. Perucca, E.; French, J.; Bialer, M. Development of new antiepileptic drugs: Challenges, incentives, and recent advances. *Lancet Neurol.* **2007**, *6*, 793–804, doi:10.1016/S1474-4422(07)70215-6.
9. Reeves, D.; Lovering, A.; Thomson, A. Therapeutic drug monitoring in the past 40 years of the Journal of Antimicrobial Chemotherapy. *J. Antimicrob. Chemother.* **2016**, *71*, 3330–3332, doi:10.1093/jac/dkw408.
10. Patsalos, P.N.; Berry, D.J.; Bourgeois, B.F.D.; Cloyd, J.C.; Glauser, T.A.; Johannessen, S.I.; Leppik, I.E.; Tomson, T.; Perucca, E. Antiepileptic drugsbest practice guidelines for therapeutic drug monitoring: A position paper by the subcommission on therapeutic drug monitoring, ILAE Commission on Therapeutic Strategies. *Epilepsia* **2008**, *49*, 1239–1276, doi:10.1111/j.1528-1167.2008.01561.x.
11. European Medicines Agency Guideline on Bioanalytical Method Validation. Available online: https://www.ema.europa.eu/en/documents/scientific-guideline/guideline-bioanalytical-method-validation_en.pdf (accessed on 16 August 2020).
12. U.S. Department of Health and Human Services Food and Drug Administration. Center for Drug Evaluation and Research (CDER) Center for Veterinary Medicine (CVM) Bioanalytical Method Validation. Guidance for Industry. Available online: https://www.fda.gov/files/drugs/published/Bioanalytical-Method-Validation-Guidance-for-Industry.pdf (accessed on 16 August 2020).
13. Ilangaratne, N.B.; Mannakkara, N.N.; Bell, G.S.; Sander, J.W. Phenobarbital: Missing in action. *Bull. World Health Organ.* **2012**, *90*, 871–871A, doi:10.2471/BLT.12.113183.
14. Oztekin, O.; Kalay, S.; Tezel, G.; Akcakus, M.; Oygur, N. Can we safely administer the recommended dose of phenobarbital in very low birth weight infants? *Childs Nerv. Syst.* **2013**, *29*, 1353–1357, doi:10.1007/s00381-013-2094-8.
15. Pacifici, M.G. Clinical Pharmacology of Phenobarbital in Neonates: Effects, Metabolism and Pharmacokinetics. *Curr. Pediatric Rev.* **2016**, *12*, 48–54, doi:10.2174/1573397111666151026223914.
16. Pokorná, P.; Šíma, M.; Vobruba, V.; Tibboel, D.; Slanař, O. Phenobarbital pharmacokinetics in neonates and infants during extracorporeal membrane oxygenation. *Perfusion* **2018**, *33*, 80–86, doi:10.1177/0267659118766444.
17. Asadi-Pooya, A.A.; Attar, A.; Moghadami, M.; Karimzadeh, I. Management of COVID-19 in people with epilepsy: Drug considerations. *Neurol. Sci.* **2020**, *41*, 2005–2011, doi:10.1007/s10072-020-04549-5.
18. Alick, S.; Doyle, A. Choosing Antiepileptic Drugs. Available online: https://practicalneurology.com/articles/2018-oct/choosing-antiepileptic-drugs?c4src=top5 (accessed on 20 August 2020).
19. Amiri Pebdani, A.; Dadfarnia, S.; Haji Shabani, A.M.; Khodadoust, S.; Talebianpoor, M.S. Modified dispersive liquid-phase microextraction based on sequential injection solidified floating organic drop combined with HPLC for the determination of phenobarbital and phenytoin. *J. Sep. Sci.* **2018**, *41*, 509–517, doi:10.1002/jssc.201701111.
20. Min, K.L.; Ryu, J.Y.; Chang, M.J. Development and clinical applications of the dried blood spot method for therapeutic drug monitoring of anti-epileptic drugs. *Basic Clin. Pharm. Toxicol.* **2019**, *125*, 215–236, doi:10.1111/bcpt.13269.
21. Velghe, S.; Deprez, S.; Stove, C.P. Fully automated therapeutic drug monitoring of anti-epileptic drugs making use of dried blood spots. *J. Chromatogr. A* **2019**, *1601*, 95–103, doi:10.1016/j.chroma.2019.06.022.
22. Velghe, S.; Stove, C.P. Volumetric absorptive microsampling as an alternative tool for therapeutic drug monitoring of first-generation anti-epileptic drugs. *Anal. Bioanal. Chem.* **2018**, *410*, 2331–2341, doi:10.1007/s00216-018-0866-4.

23. Qu, L.; Fan, Y.; Wang, W.; Ma, K.; Yin, Z. Development, validation and clinical application of an online-SPE-LC-HRMS/MS for simultaneous quantification of phenobarbital, phenytoin, carbamazepine, and its active metabolite carbamazepine 10,11-epoxide. *Talanta* **2016**, *158*, 77–88, doi:10.1016/j.talanta.2016.05.036.
24. Yin, L.; Wang, T.; Shi, M.; Zhang, Y.; Zhao, X.; Yang, Y.; Gu, J. Simultaneous determination of ten antiepileptic drugs in human plasma by liquid chromatography and tandem mass spectrometry with positive/negative ion-switching electrospray ionization and its application in therapeutic drug monitoring: Sample Preparation. *J. Sep. Sci.* **2016**, *39*, 964–972, doi:10.1002/jssc.201501067.
25. Noval, M.; Seung, H.; Armahizer, M. Evaluation of Fosphenytoin Therapeutic Drug Monitoring in the Neurocritical Care Unit. *Drugs R&D* **2020**, *20*, 17–22, doi:10.1007/s40268-019-00292-1.
26. Vossler, D.G.; Weingarten, M.; Gidal, B.E. The American Epilepsy Society Treatments Committee Summary of Antiepileptic Drugs Available in the United States of America. *Epilepsy Curr.* **2018**, *18*, 1–26, doi:10.5698/1535-7597.18.4s1.1.
27. Sills, G.J.; Rogawski, M.A. Mechanisms of action of currently used antiseizure drugs. *Neuropharmacology* **2020**, *168*, 107966, doi:10.1016/j.neuropharm.2020.107966.
28. Kang, J.; Park, Y.-S.; Kim, S.-H.; Kim, S.-H.; Jun, M.-Y. Modern Methods for Analysis of Antiepileptic Drugs in the Biological Fluids for Pharmacokinetics, Bioequivalence and Therapeutic Drug Monitoring. *Korean J. Physiol. Pharm.* **2011**, *15*, 67, doi:10.4196/kjpp.2011.15.2.67.
29. Marvanova, M. Pharmacokinetic characteristics of antiepileptic drugs (AEDs). *Ment. Health Clin.* **2016**, *6*, 8–20, doi:10.9740/mhc.2015.01.008.
30. Liu, T.; Kotha, R.R.; Jones, J.W.; Polli, J.E.; Kane, M.A. Fast liquid chromatography-tandem mass spectrometry method for simultaneous determination of eight antiepileptic drugs and an active metabolite in human plasma using polarity switching and timed selected reaction monitoring. *J. Pharm. Biomed. Anal.* **2019**, *176*, 112816, doi:10.1016/j.jpba.2019.112816.
31. Villanelli, F.; Giocaliere, E.; Malvagia, S.; Rosati, A.; Forni, G.; Funghini, S.; Shokry, E.; Ombrone, D.; Della Bona, M.L.; Guerrini, R.; et al. Dried blood spot assay for the quantification of phenytoin using Liquid Chromatography-Mass Spectrometry. *Clin. Chim. Acta* **2015**, *440*, 31–35, doi:10.1016/j.cca.2014.11.007.
32. Peat, J.; Frazee, C.; Garg, U. Quantification of Free Phenytoin by Liquid Chromatography Tandem Mass Spectrometry (LC/MS/MS). In *Clinical Applications of Mass Spectrometry in Drug Analysis*; Methods in Molecular Biology; Garg, U., Ed.; Humana Press, New York NY, USA, 2016; Volume 1383, pp. 241–246, ISBN 978-1-4939-3251-1.
33. Ochoa, J. What is the Role of Primidone in the Treatment of Epilepsy? Available online: https://www.medscape.com/answers/1187334-187110/what-is-the-role-of-primidone-in-the-treatment-of-epilepsy (accessed on 21 August 2020).
34. May, T.W.; Helmer, R.; Bien, C.G.; Brandt, C. Influence of Dose and Antiepileptic Comedication on Lacosamide Serum Concentrations in Patients With Epilepsy of Different Ages: *Ther. Drug Monit.* **2018**, *40*, 620–627, doi:10.1097/FTD.0000000000000538.
35. Hagemann, A.; Klimpel, D.; Bien, C.G.; Brandt, C.; May, T.W. Influence of dose and antiepileptic comedication on brivaracetam serum concentrations in patients with epilepsy. *Epilepsia* **2020**, *61*, doi:10.1111/epi.16500.
36. dos Santos, R.C.; Kakazu, A.K.; Santos, M.G.; Belinelli Silva, F.A.; Figueiredo, E.C. Characterization and application of restricted access carbon nanotubes in online extraction of anticonvulsant drugs from plasma samples followed by liquid chromatography analysis. *J. Chromatogr. B* **2017**, *1054*, 50–56, doi:10.1016/j.jchromb.2017.02.025.
37. D'Urso, A.; Cangemi, G.; Barco, S.; Striano, P.; D'Avolio, A.; de Grazia, U. LC-MS/MS-Based Quantification of 9 Antiepileptic Drugs From a Dried Sample Spot Device: *Ther. Drug Monit.* **2019**, *41*, 331–339, doi:10.1097/FTD.0000000000000600.
38. Patsalos, P.N.; Spencer, E.P.; Berry, D.J. Therapeutic Drug Monitoring of Antiepileptic Drugs in Epilepsy: A 2018 Update. *Ther. Drug Monit.* **2018**, *40*, 526–548, doi:10.1097/FTD.0000000000000546.
39. Hanrahan, B.; Carson, R. Ethosuximide. Available online: https://www.ncbi.nlm.nih.gov/books/NBK544244/ (accessed on 21 August 2020).
40. Wu, Y.-J.; Li, Y.-S.; Tseng, W.-L.; Lu, C.-Y. Microextraction combined with microderivatization for drug monitoring and protein modification analysis from limited blood volume using mass spectrometry. *Anal. Bioanal. Chem.* **2018**, *410*, 7405–7414, doi:10.1007/s00216-018-1349-3.

41. Baldelli, S.; Cattaneo, D.; Giodini, L.; Baietto, L.; Di Perri, G.; D'Avolio, A.; Clementi, E. Development and validation of a HPLC-UV method for the quantification of antiepileptic drugs in dried plasma spots. *Clin. Chem. Lab. Med.* **2015**, *53*, doi:10.1515/cclm-2014-0472.
42. Dao, K.; Thoueille, P.; Decosterd, L.A.; Mercier, T.; Guidi, M.; Bardinet, C.; Lebon, S.; Choong, E.; Castang, A.; Guittet, C.; et al. Sultiame pharmacokinetic profile in plasma and erythrocytes after single oral doses: A pilot study in healthy volunteers. *Pharm. Res Perspect* **2020**, *8*, doi:10.1002/prp2.558.
43. Aledo-Serrano, A.; Gil-Nagel, A. Anticonvulsant Agents: Carbamazepine, Oxcarbazepine, and Eslicarbazepine Acetate. In *NeuroPsychopharmacotherapy*; Riederer, P., Laux, G., Mulsant, B., Le, W., Nagatsu, T., Eds.; Springer International Publishing: Cham, Switzerland, 2020; pp. 1–8, ISBN 978-3-319-56015-1.
44. Greenwood, J.; Valdes, J. Perampanel (Fycompa): A Review of Clinical Efficacy and Safety in Epilepsy. *P T* **2016**, *41*, 683–698.
45. Jacob, S.; Nair, A.B. An Updated Overview on Therapeutic Drug Monitoring of Recent Antiepileptic Drugs. *Drugs R&D* **2016**, *16*, 303–316, doi:10.1007/s40268-016-0148-6.
46. Bahmaei, M.; Khalilian, F.; Mashayekhi, H.A. Determination of Carbamazepine in Biological Samples Using Ultrasound-Assisted Emulsification Micro-extraction and Gas Chromatography. *J. Chem. Health Risks* **2015**, *5*, doi:10.22034/jchr.2018.544093.
47. Samadi, A.; Khoubnasabjafari, M.; Barzegar, M.; Sadeghvand, S.; Shiva, S.; Jouyban, A. Simultaneous Determination of Phenobarbital, Phenytoin, Carbamazepine and Carbamazepine-10,11-epoxide in Plasma of Epileptic Patients. *Pharm. Sci.* **2019**, *25*, 345–351, doi:10.15171/PS.2019.43.
48. Ezzeldin, E.; Shahat, A.A.; Basudan, O.A. Development and Validation of an HPLC Method for the Determination of Carbamazepine in Human Plasma. *Life Sci. J.* **2013**, *10*, 2159–2163.
49. Mohamed, F.A.; Ali, M.F.B.; Rageh, A.H.; Mostafa, A.M. A highly sensitive HPTLC method for estimation of oxcarbazepine in two binary mixtures with two metabolically related antiepileptic drugs: Application to pharmaceutical and biological samples. *Microchem. J.* **2019**, *146*, 414–422, doi:10.1016/j.microc.2019.01.031.
50. Behbahani, M.; Najafi, F.; Bagheri, S.; Bojdi, M.K.; Salarian, M.; Bagheri, A. Application of surfactant assisted dispersive liquid–liquid microextraction as an efficient sample treatment technique for preconcentration and trace detection of zonisamide and carbamazepine in urine and plasma samples. *J. Chromatogr. A* **2013**, *1308*, 25–31, doi:10.1016/j.chroma.2013.07.088.
51. Budikayanti, A.; Chaliana, C.; Louisa, M.; Setiabudy, R. Development and validation of carbamazepine plasma concentrations measurement and its application on epilepsy patients. *Int. J. Pharm. Pharm. Sci.* **2017**, *9*, 87, doi:10.22159/ijpps.2017v9i9.19402.
52. Farouk, F.; ElKady, E.F.; Azzazy, H.M.E. Simultaneous UPLC-MS/MS determination of antiepileptic agents for dose adjustment. *Biomed. Chromatogr.* **2017**, *31*, e3921, doi:10.1002/bmc.3921.
53. Andonie, D.; Gáll, Z.; Bosa, P.; Dogaru, M.T.; Vancea, S. Simultaneous Determination of Carbamazepine and Carbamazepine-10,11-epoxide in Different Biological Matrices by LC-MS/MS. *J. Interdiscip. Med.* **2017**, *2*, 211–218, doi:10.1515/jim-2017-0075.
54. Wang, L.; Wang, J.; Zhang, J.; Jiang, Q.; Zhao, L.; Zhang, T. Simultaneous determination of topiramate, carbamazepine, oxcarbazepine and its major metabolite in human plasma by SFC-ESI-MS/MS with polarity switching: Application to therapeutic drug monitoring. *Arab. J. Chem.* **2019**, *12*, 4775–4783, doi:10.1016/j.arabjc.2016.09.016.
55. Ghafghazi, S.; Moini Zanjani, T.; Vosough, M.; Sabetkasaei, M. Interference-free Determination of Carbamazepine in Human Serum Using High Performance Liquid Chromatography: A Comprehensive Research with Three-way Calibration Methods. *Iran. J. Pharm. Res.* **2017**, *16*, 120–131.
56. Sultana, N.; Arayne, M.S.; Ali, S.N. An Ultra-sensitive LC Method for the Simultaneous Determination of Paracetamol, Carbamazepine, Losartan and Ciprofloxacin in Bulk Drug, Pharmaceutical Formulation and Human Serum by Programming the Detector. *AJAC* **2013**, *4*, 24–33, doi:10.4236/ajac.2013.41004.
57. Jiang, W.; Xia, T.; Yun, Y.; Li, M.; Zhang, F.; Gao, S.; Chen, W. UHPLC-MS/MS method for simultaneous determination of carbamazepine and its seven major metabolites in serum of epileptic patients. *J. Chromatogr. B* **2019**, *1108*, 17–24, doi:10.1016/j.jchromb.2018.12.016.

58. Feriduni, B.; Farajzadeh, M.A.; Jouyban, A. Determination of Two Antiepileptic Drugs in Urine by Homogenous Liquid-Liquid Extraction Performed in A Narrow Tube Combined with Dispersive Liquid-liquid Microextraction Followed by Gas Chromatography-flame Ionization Detection. *Iran. J. Pharm. Res.* **2019**, doi:10.22037/ijpr.2019.1100635.
59. Dziurkowska, E.; Wesolowski, M. Deproteinization as a Rapid Method of Saliva Purification for the Determination of Carbamazepine and Carbamazepine-10,11 Epoxide. *J. Clin. Med.* **2020**, *9*, 915, doi:10.3390/jcm9040915.
60. Carvalho, J.; Rosado, T.; Barroso, M.; Gallardo, E. Determination of Antiepileptic Drugs Using Dried Saliva Spots. *J. Anal. Toxicol.* **2019**, *43*, 61–71, doi:10.1093/jat/bky064.
61. Hatefi, A.; Rahimpour, E.; Khoubnasabjafari, M.; Edalat, M.; Jouyban-Gharamaleki, V.; Alvani-Alamdari, S.; Nokhodchi, A.; Pournaghi-Azar, M.H.; Jouyban, A. A single-shot diagnostic platform based on copper nanoclusters coated with cetyl trimethylammonium bromide for determination of carbamazepine in exhaled breath condensate. *Microchim. Acta* **2019**, *186*, 194, doi:10.1007/s00604-019-3278-z.
62. Khoubnasabjafari, M.; Rahimpour, E.; Jouyban, A. Exhaled breath condensate as an alternative sample for drug monitoring. *Bioanalysis* **2018**, *10*, 61–64, doi:10.4155/bio-2017-0205.
63. Kong, S.T.; Lim, S.-H.; Lee, W.B.; Kumar, P.K.; Wang, H.Y.S.; Ng, Y.L.S.; Wong, P.S.; Ho, P.C. Clinical Validation and Implications of Dried Blood Spot Sampling of Carbamazepine, Valproic Acid and Phenytoin in Patients with Epilepsy. *PLoS ONE* **2014**, *9*, e108190, doi:10.1371/journal.pone.0108190.
64. Linder, C.; Neideman, M.; Wide, K.; von Euler, M.; Gustafsson, L.L.; Pohanka, A. Dried Blood Spot Self-Sampling by Guardians of Children With Epilepsy Is Feasible: Comparison With Plasma for Multiple Antiepileptic Drugs. *Ther. Drug Monit.* **2019**, *41*, 509–518, doi:10.1097/FTD.0000000000000605.
65. Ferreira, A.; Rodrigues, M.; Oliveira, P.; Francisco, J.; Fortuna, A.; Rosado, L.; Rosado, P.; Falcão, A.; Alves, G. Liquid chromatographic assay based on microextraction by packed sorbent for therapeutic drug monitoring of carbamazepine, lamotrigine, oxcarbazepine, phenobarbital, phenytoin and the active metabolites carbamazepine-10,11-epoxide and licarbazepine. *J. Chromatogr. B* **2014**, *971*, 20–29, doi:10.1016/j.jchromb.2014.09.010.
66. Mano, Y. LC–MS-MS Determination of Oxcarbazepine and an Active Metabolite in Human Plasma for Clinical Application. *J. Chromatogr. Sci.* **2018**, *56*, 687–694, doi:10.1093/chromsci/bmy040.
67. Shibata, M.; Hashi, S.; Nakanishi, H.; Masuda, S.; Katsura, T.; Yano, I. Detection of 22 antiepileptic drugs by ultra-performance liquid chromatography coupled with tandem mass spectrometry applicable to routine therapeutic drug monitoring: UPLC-MS/MS method for detection of 22 antiepileptic drugs. *Biomed. Chromatogr.* **2012**, *26*, 1519–1528, doi:10.1002/bmc.2726.
68. May, T.W.; Korn-Merker, E.; Rambeck, B. Clinical Pharmacokinetics of Oxcarbazepine: *Clin. Pharmacokinet.* **2003**, *42*, 1023–1042, doi:10.2165/00003088-200342120-00002.
69. Gierbolini, J.; Giarratano, M.; Benbadis, S.R. Carbamazepine-related antiepileptic drugs for the treatment of epilepsy—A comparative review. *Expert Opin. Pharmacother.* **2016**, *17*, 885–888, doi:10.1517/14656566.2016.1168399.
70. Serralheiro, A.; Alves, G.; Fortuna, A.; Rocha, M.; Falcão, A. First HPLC–UV method for rapid and simultaneous quantification of phenobarbital, primidone, phenytoin, carbamazepine, carbamazepine-10,11-epoxide, 10,11-trans-dihydroxy-10,11-dihydrocarbamazepine, lamotrigine, oxcarbazepine and licarbazepine in human plasma. *J. Chromatogr. B* **2013**, *925*, 1–9, doi:10.1016/j.jchromb.2013.02.026.
71. Jin, S.; Zhao, Q.; Zhang, D.; Zhao, Z.; Mei, S. Development and validation of an improved HPLC-UV method for simultaneous determination of lamotrigine and oxcarbazepine and its active metabolite 10,11-dihydro-10-hydroxycarbazepine in human blood plasma and comparison with an UHPLC-MS/MS method. *J. Anal. Sci. Technol.* **2019**, *10*, 36, doi:10.1186/s40543-019-0198-9.
72. Fortuna, A.; Sousa, J.; Alves, G.; Falcão, A.; Soares-da-Silva, P. Development and validation of an HPLC-UV method for the simultaneous quantification of carbamazepine, oxcarbazepine, eslicarbazepine acetate and their main metabolites in human plasma. *Anal. Bioanal. Chem.* **2010**, *397*, 1605–1615, doi:10.1007/s00216-010-3673-0.

73. Loureiro, A.I.; Fernandes-Lopes, C.; Wright, L.C.; Soares-da-Silva, P. Development and validation of an enantioselective liquid-chromatography/tandem mass spectrometry method for the separation and quantification of eslicarbazepine acetate, eslicarbazepine, R-licarbazepine and oxcarbazepine in human plasma. *J. Chromatogr. B* **2011**, *879*, 2611–2618, doi:10.1016/j.jchromb.2011.07.019.
74. Dupouey, J.; Doudka, N.; Belo, S.; Blin, O.; Guilhaumou, R. Simultaneous determination of four antiepileptic drugs in human plasma samples using an ultra-high-performance liquid chromatography tandem mass spectrometry method and its application in therapeutic drug monitoring: Simultaneous quantification of antiepileptic drugs by LC-MS/MS. *Biomed. Chromatogr.* **2016**, *30*, 2053–2060, doi:10.1002/bmc.3789.
75. Palte, M.J.; Basu, S.S.; Dahlin, J.L.; Gencheva, R.; Mason, D.; Jarolim, P.; Petrides, A.K. Development and Validation of an Ultra-Performance Liquid Chromatography–Tandem Mass Spectrometry Method for the Concurrent Measurement of Gabapentin, Lamotrigine, Levetiracetam, Monohydroxy Derivative of Oxcarbazepine, and Zonisamide Concentrations in Serum in a Clinical Setting: *Ther. Drug Monit.* **2018**, *40*, 469–476, doi:10.1097/FTD.0000000000000516.
76. Rani, S.; Malik, A.K. A novel microextraction by packed sorbent-gas chromatography procedure for the simultaneous analysis of antiepileptic drugs in human plasma and urine: Sample Preparation. *J. Sep. Sci.* **2012**, *35*, 2970–2977, doi:10.1002/jssc.201200439.
77. Málaga, I.; Sánchez-Carpintero, R.; Roldán, S.; Ramos-Lizana, J.; García-Peñas, J.J. New antiepileptic drugs in pediatrics. *An. Pediatría* **2019**, *91*, 415e1–415e10, doi:10.1016/j.anpedi.2019.09.008.
78. Lattanzi, S.; Brigo, F.; Cagnetti, C.; Verrotti, A.; Zaccara, G.; Silvestrini, M. Eslicarbazepine acetate in the treatment of adults with partial-onset epilepsy: An evidence-based review of efficacy, safety and place in therapy. *Core Evid.* **2018**, *13*, 21–31, doi:10.2147/CE.S142858.
79. Bialer, M.; Soares-da-Silva, P. Pharmacokinetics and drug interactions of eslicarbazepine acetate: Pharmacokinetics and Drug Interactions of ESL. *Epilepsia* **2012**, *53*, 935–946, doi:10.1111/j.1528-1167.2012.03519.x.
80. Galiana, G.L.; Gauthier, A.C.; Mattson, R.H. Eslicarbazepine Acetate: A New Improvement on a Classic Drug Family for the Treatment of Partial-Onset Seizures. *Drugs R&D* **2017**, *17*, 329–339, doi:10.1007/s40268-017-0197-5.
81. Deeb, S.; McKeown, D.A.; Torrance, H.J.; Wylie, F.M.; Logan, B.K.; Scott, K.S. Simultaneous Analysis of 22 Antiepileptic Drugs in Postmortem Blood, Serum and Plasma Using LC–MS-MS with a Focus on Their Role in Forensic Cases. *J. Anal. Toxicol.* **2014**, *38*, 485–494, doi:10.1093/jat/bku070.
82. Tolbert, D.; Larsen, F. A Comprehensive Overview of the Clinical Pharmacokinetics of Clobazam. *J. Clin. Pharmacol.* **2019**, *59*, 7–19, doi:10.1002/jcph.1313.
83. Déglon, J.; Versace, F.; Lauer, E.; Widmer, C.; Mangin, P.; Thomas, A.; Staub, C. Rapid LC–MS/MS quantification of the major benzodiazepines and their metabolites on dried blood spots using a simple and cost-effective sample pretreatment. *Bioanalysis* **2012**, *4*, 1337–1350, doi:10.4155/bio.12.42.
84. Diniz, M.E.R.; Dias, N.L.; Paulo, B.P.; Andrade, F.V.; Mateo, E.C.; Ferreira, A.C.S. Development and validation of method for the determination of the benzodiazepines clonazepam, clobazam and N-Desmethylclobazam in serum by LC-MS/MS and its application in clinical routine. *Br. J. Anal. Chem.* **2017**, *4*, 8–16.
85. Mikayelyan, A.; Aleksanyan, A.; Sargsyan, M.; Gevorgyan, A.; Zakaryan, H.; Harutyunyan, A.; Zhamharyan, L.; Armoudjian, Y.; Margaryan, T. Protein precipitation method for determination of clobazam and *N*-desmethylclobazam in human plasma by LC–MS/MS. *Biomed. Chromatogr.* **2020**, *34*, doi:10.1002/bmc.4710.
86. Winblad, B. Piracetam: A Review of Pharmacological Properties and Clinical Uses. *CNS Drug Rev.* **2006**, *11*, 169–182, doi:10.1111/j.1527-3458.2005.tb00268.x.
87. Sirotina, I.V.; Aleksandrova, Z.D.; Bogatyreva, N.V.; Gusel', V.A.; Smirnov, D.P.; Khodov, D.A.; Grandilevskaia, O.L. The results of the therapeutic monitoring of piracetam in parturients and newborn infants. *Eksp. Klin. Farm.* **1992**, *55*, 53–56.
88. Curticapean, A.; Imre, S. New validated method for piracetam HPLC determination in human plasma. *J. Biochem. Biophys. Methods* **2007**, *69*, 273–281, doi:10.1016/j.jbbm.2006.06.001.
89. Yeh, H.-H.; Yang, Y.-H.; Ko, J.-Y.; Chen, S.-H. Rapid determination of piracetam in human plasma and cerebrospinal fluid by micellar electrokinetic chromatography with sample direct injection. *J. Chromatogr. A* **2006**, *1120*, 27–34, doi:10.1016/j.chroma.2005.11.071.

90. Siddiqui, F.A.; Sher, N.; Shafi, N.; Wafa Sial, A.; Ahmad, M.; Mehjebeen; Naseem, H. Development of New Method for Simultaneous Analysis of Piracetam and Levetiracetam in Pharmaceuticals and Biological Fluids: Application in Stability Studies. *BioMed Res. Int.* **2014**, *2014*, 1–8, doi:10.1155/2014/758283.
91. Mendes, G.D.; Zaffalon, G.T.; Silveira, A.S.; Ramacciato, J.C.; Motta, R.H.L.; Gagliano-Jucá, T.; Lopes, A.G.; de Almeida Magalhães, J.C.; De Nucci, G. Assessment of pharmacokinetic interaction between piracetam and l-carnitine in healthy subjects: Piracetam and L-carnitine pharmacokinetic interaction study. *Biomed. Chromatogr.* **2016**, *30*, 536–542, doi:10.1002/bmc.3579.
92. Doheny, M.H.; O'Connell, M.T.; Patsalos, P.N. A High-performance Liquid-chromatographic Microanalytical Procedure for the Rapid Estimation of Piracetam in Plasma or Cerebrospinal Fluid. *J. Pharm. Pharmacol.* **1996**, *48*, 514–516, doi:10.1111/j.2042-7158.1996.tb05964.x.
93. Louchahi, K.; Tod, M.; Bonnardel, P.; Petitjean, O. Determination of piracetam in human plasma and urine by liquid chromatography. *J. Chromatogr. B Biomed. Sci. Appl.* **1995**, *663*, 385–389, doi:10.1016/0378-4347(94)00448-E.
94. Klein, P.; Diaz, A.; Gasalla, T.; Whitesides, J. A review of the pharmacology and clinical efficacy of brivaracetam. *Clin. Pharmacol. Adv. Appl.* **2018**, *10*, 1–22, doi:10.2147/CPAA.S114072.
95. Steinhoff, B.J.; Staack, A.M. Levetiracetam and brivaracetam: A review of evidence from clinical trials and clinical experience. *Adv. Neurol. Disord.* **2019**, *12*, 175628641987351, doi:10.1177/1756286419873518.
96. Sourbron, J.; Chan, H.; Wammes-van der Heijden, E.A.; Klarenbeek, P.; Wijnen, B.F.M.; de Haan, G.-J.; van der Kuy, H.; Evers, S.; Majoie, M. Review on the relevance of therapeutic drug monitoring of levetiracetam. *Seizure* **2018**, *62*, 131–135, doi:10.1016/j.seizure.2018.09.004.
97. Moseley, B.D.; Chanteux, H.; Nicolas, J.-M.; Laloyaux, C.; Gidal, B.; Stockis, A. A review of the drug−drug interactions of the antiepileptic drug brivaracetam. *Epilepsy Res.* **2020**, *163*, 106327, doi:10.1016/j.eplepsyres.2020.106327.
98. Howard, P.; Remi, J.; Remi, C.; Charlesworth, S.; Whalley, H.; Bhatia, R.; Hitchens, M.; Mihalyo, M.; Wilcock, A. Levetiracetam. *J. Pain Symptom Manag.* **2018**, *56*, 645–649, doi:10.1016/j.jpainsymman.2018.07.012.
99. Mohamed, S.; Riva, R.; Contin, M. Development and Validation of an UHPLC-MS/MS Assay for the Therapeutic Monitoring of Brivaracetam Plasma Concentrations in Patients with Epilepsy: *Ther. Drug Monit.* **2020**, *42*, 445–451, doi:10.1097/FTD.0000000000000726.
100. Yeap, L.-L.; Lo, Y.-L. Rapid and Simultaneous Quantification of Levetiracetam and Its Carboxylic Metabolite in Human Plasma by Liquid Chromatography Tandem Mass Spectrometry. *PLoS ONE* **2014**, *9*, e111544, doi:10.1371/journal.pone.0111544.
101. El-Yazbi, A.F.; Wagih, M.M.; Ibrahim, F.; Barary, M.A. Spectrofluorimetric Determination of Topiramate and Levetiracetam as Single Components in Tablet Formulations and in Human Plasma and Simultaneous Fourth Derivative Synchronous Fluorescence Determination of their Co-Adminstered Mixture in Human Plasma. *J. Fluoresc.* **2016**, *26*, 1225–1238, doi:10.1007/s10895-016-1810-7.
102. Iqbal, M.; Ezzeldin, E.; Al-Rashood, K.A. UPLC–MS/MS assay for identification and quantification of brivaracetam in plasma sample: Application to pharmacokinetic study in rats. *J. Chromatogr. B* **2017**, *1060*, 63–70, doi:10.1016/j.jchromb.2017.05.039.
103. Mohamed, F.A.; Ali, M.F.B.; Rageh, A.H.; Mostafa, A.M. Highly sensitive UHPLC–DAD method for simultaneous determination of two synergistically acting antiepileptic drugs; levetiracetam and lacosamide: Application to pharmaceutical tablets and human urine. *Biomed. Chromatogr.* **2019**, *33*, doi:10.1002/bmc.4554.
104. Sim, J.; Kim, E.; Yang, W.; Woo, S.; In, S. An LC–MS/MS method for the simultaneous determination of 15 antipsychotics and two metabolites in hair and its application to rat hair. *Forensic Sci. Int.* **2017**, *274*, 91–98, doi:10.1016/j.forsciint.2017.01.001.
105. Gonçalves, J.; Alves, G.; Bicker, J.; Falcão, A.; Fortuna, A. Development and full validation of an innovative HPLC-diode array detection technique to simultaneously quantify lacosamide, levetiracetam and zonisamide in human plasma. *Bioanalysis* **2018**, *10*, 541–557, doi:10.4155/bio-2017-0199.
106. Pucci, V.; Bugamelli, F.; Mandrioli, R.; Ferranti, A.; Kenndler, E.; Raggi, M.A. High-performance liquid chromatographic determination of Levetiracetam in human plasma: Comparison of different sample clean-up procedures. *Biomed. Chromatogr.* **2004**, *18*, 37–44, doi:10.1002/bmc.289.

107. Bourgogne, E.; Culot, B.; Dell'Aiera, S.; Chanteux, H.; Stockis, A.; Nicolas, J.-M. Off-line solid phase extraction and liquid chromatography-tandem mass spectrometry method for the quantitation of brivaracetam acid metabolites: Method validation and application to in vitro metabolism assays. *J. Chromatogr. B* **2018**, *1086*, 138–145, doi:10.1016/j.jchromb.2018.04.018.
108. Engelbrecht, L.; Grobler, C.J.; Rheeders, M. A simple and cost-effective HPLC-UV method for the detection of levetiracetam in plasma/serum of patients with epilepsy. *Biomed. Chromatogr.* **2017**, *31*, e3969, doi:10.1002/bmc.3969.
109. Mendoza Aguilera, M.; Bellés Medall, M.D.; Álvarez Martín, T.; Pascual Marmaneu, Ó.; Liñana Granell, C.; Ferrando Piqueres, R. Therapeutic drug monitoring of levetiracetam in daily clinical practice: High-performance liquid chromatography versus immunoassay. *Eur. J. Hosp. Pharm.* **2020**, *27*, e2–e6, doi:10.1136/ejhpharm-2018-001616.
110. Kalaria, S.N.; Dahmane, E.; Armahizer, M.; McCarthy, P.; Gopalakrishnan, M. Development and validation of a HPLC-UV assay for quantification of levetiracetam concentrations in critically ill patients undergoing continuous renal replacement therapy. *Biomed. Chromatogr.* **2018**, *32*, e4257, doi:10.1002/bmc.4257.
111. Olah, E.; Bacsoi, G.; Fekete, J.; Sharma, V.K. Determination of ng/mL Levetiracetam using Ultra-High-Performance Liquid Chromatography-Photodiode Absorbance. *J. Chromatogr. Sci.* **2012**, *50*, 253–258, doi:10.1093/chromsci/bmr053.
112. Reineks, E.Z.; Lawson, S.E.; Lembright, K.E.; Wang, S. Performance Characteristics of a New Levetiracetam Immunoassay and Method Comparison With a High-Performance Liquid Chromatography Method: *Ther. Drug Monit.* **2011**, *33*, 124–127, doi:10.1097/FTD.0b013e3182048e05.
113. Bae, E.-K.; Lee, J.; Shin, J.-W.; Moon, J.; Lee, K.-J.; Shin, Y.-W.; Kim, T.-J.; Shin, D.; Jang, I.-J.; Lee, S.K. Factors influencing topiramate clearance in adult patients with epilepsy: A population pharmacokinetic analysis. *Seizure* **2016**, *37*, 8–12, doi:10.1016/j.seizure.2016.02.002.
114. Milosheska, D.; Vovk, T.; Grabnar, I.; Roškar, R. Simple and sensitive high performance liquid chromatography method with fluorescence detection for therapeutic drug monitoring of topiramate. *ACSi* **2015**, *62*, 411–419, doi:10.17344/acsi.2014.382.
115. Milosheska, D.; Roškar, R. A novel LC–MS/MS method for the simultaneous quantification of topiramate and its main metabolites in human plasma. *J. Pharm. Biomed. Anal.* **2017**, *138*, 180–188, doi:10.1016/j.jpba.2017.02.003.
116. Hahn, R.Z.; Antunes, M.V.; Costa Arnhold, P.; Andriguetti, N.B.; Verza, S.G.; Linden, R. Determination of topiramate in dried blood spots using single-quadrupole gas chromatography–mass spectrometry after flash methylation with trimethylanilinium hydroxide. *J. Chromatogr. B* **2017**, *1046*, 131–137, doi:10.1016/j.jchromb.2017.01.047.
117. Ni, Y.; Zhou, Y.; Xu, M.; He, X.; Li, H.; Haseeb, S.; Chen, H.; Li, W. Simultaneous determination of phentermine and topiramate in human plasma by liquid chromatography–tandem mass spectrometry with positive/negative ion-switching electrospray ionization and its application in pharmacokinetic study. *J. Pharm. Biomed. Anal.* **2015**, *107*, 444–449, doi:10.1016/j.jpba.2015.01.035.
118. Ishikawa, A.A.; da Silva, R.M.; Santos, M.S.F.; da Costa, E.T.; Sakamoto, A.C.; Carrilho, E.; de Gaitani, C.M.; Garcia, C.D. Determination of topiramate by capillary electrophoresis with capacitively-coupled contactless conductivity detection: A powerful tool for therapeutic monitoring in epileptic patients. *Electrophoresis* **2018**, *39*, 2598–2604, doi:10.1002/elps.201800046.
119. French, J.; Smith, M.; Faught, E.; Brown, L. Practice advisory: The use of felbamate in the treatment of patients with intractable epilepsy: Report of the Quality Standards Subcommittee of the American Academy of Neurology and the American Epilepsy Society. *Neurology* **1999**, *52*, 1540–1540, doi:10.1212/WNL.52.8.1540.
120. Harty, T.P.; Rogawski, M.A. Felbamate block of recombinant N-methyl-d-aspartate receptors: Selectivity for the NR2B subunit. *Epilepsy Res.* **2000**, *39*, 47–55, doi:10.1016/S0920-1211(99)00108-4.
121. Krasowski, M.D. Therapeutic Drug Monitoring of the Newer Anti-Epilepsy Medications. *Pharmaceuticals* **2010**, *3*, 1909–1935, doi:10.3390/ph3061909.
122. Gur, P.; Poklis, A.; Saady, J.; Costantino, A. Chromatographic Procedures for the Determination of Felbamate in Serum. *J. Anal. Toxicol.* **1995**, *19*, 499–503, doi:10.1093/jat/19.6.499.

123. Contin, M.; Balboni, M.; Callegati, E.; Candela, C.; Albani, F.; Riva, R.; Baruzzi, A. Simultaneous liquid chromatographic determination of lamotrigine, oxcarbazepine monohydroxy derivative and felbamate in plasma of patients with epilepsy. *J. Chromatogr. B* **2005**, *828*, 113–117, doi:10.1016/j.jchromb.2005.09.009.
124. Chateauvieux, S.; Morceau, F.; Dicato, M.; Diederich, M. Molecular and Therapeutic Potential and Toxicity of Valproic Acid. *J. Biomed. Biotechnol.* **2010**, *2010*, 1–18, doi:10.1155/2010/479364.
125. Ghodke-Puranik, Y.; Thorn, C.F.; Lamba, J.K.; Leeder, J.S.; Song, W.; Birnbaum, A.K.; Altman, R.B.; Klein, T.E. Valproic acid pathway: Pharmacokinetics and pharmacodynamics. *Pharm. Genom.* **2013**, *23*, 236–241, doi:10.1097/FPC.0b013e32835ea0b2.
126. Perucca, E. Clinically relevant drug interactions with antiepileptic drugs. *Br. J. Clin. Pharm.* **2006**, *61*, 246–255, doi:10.1111/j.1365-2125.2005.02529.x.
127. Feriduni, B.; Barzegar, M.; Sadeghvand, S.; Shiva, S.; Khoubnasabjafari, M.; Jouyban, A. Determination of valproic acid and 3-heptanone in plasma using air-assisted liquid-liquid microextraction with the assistance of vortex: Application in the real samples. *Bioimpacts* **2019**, *9*, 105–113, doi:10.15171/bi.2019.14.
128. Fazeli-Bakhtiyari, R.; Panahi-Azar, V.; Sorouraddin, M.H.; Jouyban, A. Determination of valproic acid in human plasma using dispersive liquid-liquid microextraction followed by gas chromatography-flame ionization detection. *Iran. J. Basic Med. Sci.* **2015**, *18*, 979–988.
129. Mostafa, M.S.; Elshafie, H.S.; Ghaleb, S. A rapid and simple procedure for monitoring valproic acid by gas chromatography. *J. Biol. Res.* **2018**, *90*, doi:10.4081/jbr.2017.6359.
130. Harahap, Y.; Ikhsan, M. Determination of Valproic Acid without Derivatization in Human Plasma using High Performance Liquid Chromatography-Photodiode Array. *J. Glob. Pharma Technol.* **2017**, *6*, 82–89.
131. Dwivedi, R.; Gupta, Y.K.; Singh, M.; Joshi, R.; Tiwari, P.; Kaleekal, T.; Tripathi, M. Correlation of saliva and serum free valproic acid concentrations in persons with epilepsy. *Seizure* **2015**, *25*, 187–190, doi:10.1016/j.seizure.2014.10.010.
132. Lu, H.; Su, C.; Yin, L.; Gu, L.; Gu, J.; Chen, X. Liquid chromatography–tandem mass spectrometry method for simultaneous determination of valproic acid and its ene-metabolites in epilepsy patient plasma. *J. Pharm. Anal.* **2016**, *6*, 112–116, doi:10.1016/j.jpha.2015.11.006.
133. Zhao, M.; Zhang, T.; Li, G.; Qiu, F.; Sun, Y.; Zhao, L. Simultaneous Determination of Valproic Acid and Its Major Metabolites by UHPLC-MS/MS in Chinese Patients: Application to Therapeutic Drug Monitoring. *J. Chromatogr. Sci.* **2016**, *55*, doi:10.1093/chromsci/bmw199.
134. Wang, Z.; Yun, Y.; Xie, X.; You, C.; Miao, H.; Zhang, F.; Gao, S.; Chen, W. Comparison of LC-MS/MS vs chemiluminescent microparticle immunoassay in measuring the valproic acid concentration in plasma of epilepsy patients in a new perspective. *J. Clin. Lab. Anal.* **2018**, *32*, e22157, doi:10.1002/jcla.22157.
135. Zhao, M.; Li, G.; Qiu, F.; Sun, Y.; Xu, Y.; Zhao, L. Development and Validation of a Simple and Rapid UPLC–MS Assay for Valproic Acid and Its Comparison With Immunoassay and HPLC Methods: *Ther. Drug Monit.* **2016**, *38*, 246–252, doi:10.1097/FTD.0000000000000256.
136. Gajcy, K.; Lochynski, S.; Librowski, T. A Role of GABA Analogues in the Treatment of Neurological Diseases. *Curr. Med. Chem.* **2010**, *17*, 2338–2347, doi:10.2174/092986710791698549.
137. Greenblatt, H.K.; Greenblatt, D.J. Gabapentin and Pregabalin for the Treatment of Anxiety Disorders. *Clin. Pharmacol. Drug Dev.* **2018**, *7*, 228–232, doi:10.1002/cpdd.446.
138. Bockbrader, H.N.; Wesche, D.; Miller, R.; Chapel, S.; Janiczek, N.; Burger, P. A Comparison of the Pharmacokinetics and Pharmacodynamics of Pregabalin and Gabapentin: *Clin. Pharmacokinet.* **2010**, *49*, 661–669, doi:10.2165/11536200-000000000-00000.
139. De La Vega, H.; Fox, K.; Pardi, J.; Santiago-Tirado, W.; Cooper, G. Validation of a High-throughput Screening and Quantification Method for the Determination of Gabapentinoids in Blood Using a Combination of LC-TOF-MS and LC-MS-MS. *J. Anal. Toxicol.* **2019**, *43*, 696–702, doi:10.1093/jat/bkz070.
140. Nahar, L.; Smith, A.; Patel, R.; Andrews, R.; Paterson, S. Validated Method for the Screening and Quantification of Baclofen, Gabapentin and Pregabalin in Human Post-Mortem Whole Blood Using Protein Precipitation and Liquid Chromatography–Tandem Mass Spectrometry. *J. Anal. Toxicol.* **2017**, *41*, 441–450, doi:10.1093/jat/bkx019.
141. Hložek, T.; Bursová, M.; Coufal, P.; Čabala, R.; Gabapentin, Pregabalin and Vigabatrin Quantification in Human Serum by GC–MS After Hexyl Chloroformate Derivatization. *J. Anal. Toxicol.* **2016**, *40*, 749-753, doi:10.1093/jat/bkw070.

142. Sadones, N.; Van Bever, E.; Van Bortel, L.; Lambert, W.E.; Stove, C.P. Dried blood spot analysis of gabapentin as a valid alternative for serum: A bridging study. *J. Pharm. Biomed. Anal.* **2017**, *132*, 72–76, doi:10.1016/j.jpba.2016.09.036.
143. Yoshikawa, N.; Naito, T.; Yagi, T.; Kawakami, J. A Validated Fluorometric Method for the Rapid Determination of Pregabalin in Human Plasma Applied to Patients With Pain. *Ther. Drug Monit.* **2016**, *38*, 628–633, doi:10.1097/FTD.0000000000000325.
144. Dwivedi, J.; Namdev, K.K.; Chilkoti, D.C.; Verma, S.; Sharma, S. An Improved LC-ESI-MS/MS Method to Quantify Pregabalin in Human Plasma and Dry Plasma Spot for Therapeutic Monitoring and Pharmacokinetic Applications: *Ther. Drug Monit.* **2018**, *40*, 610–619, doi:10.1097/FTD.0000000000000541.
145. Kostić, N.; Dotsikas, Y.; Jović, N.; Stevanović, G.; Malenović, A.; Medenica, M. Quantitation of pregabalin in dried blood spots and dried plasma spots by validated LC–MS/MS methods. *J. Pharm. Biomed. Anal.* **2015**, *109*, 79–84, doi:10.1016/j.jpba.2015.02.023.
146. Bauer, J. Tiagabine: Efficacy and safety in partial seizures—Current status. *Neuropsychiatr. Dis. Treat.* **2008**, *2008*, 731–736, doi:10.2147/NDT.S833.
147. Tolman, J.A.; Faulkner, M.A. Vigabatrin: A comprehensive review of drug properties including clinical updates following recent FDA approval. *Expert Opin. Pharmacother.* **2009**, *10*, 3077–3089, doi:10.1517/14656560903451690.
148. Police, A.; Shankar, V.K.; Narasimha Murthy, S. RP-HPLC method for simultaneous estimation of vigabatrin, gamma-aminobutyric acid and taurine in biological samples. *J. Chromatogr. B* **2018**, *1076*, 44–53, doi:10.1016/j.jchromb.2018.01.010.
149. Duhamel, P.; Ounissi, M.; Le Saux, T.; Bienayme, H.; Chiron, C.; Jullien, V. Determination of the R (−) and S (+)-enantiomers of vigabatrin in human plasma by ultra-high-performance liquid chromatography and tandem mass-spectrometry. *J. Chromatogr. B* **2017**, *1070*, 31–36, doi:10.1016/j.jchromb.2017.10.037.
150. Yasam, V.R.; Jakki, S.L.; Senthil, V.; Eswaramoorthy, M.; Shanmuganathan, S.; Arjunan, K.; Nanjan, M. A pharmacological overview of lamotrigine for the treatment of epilepsy. *Expert Rev. Clin. Pharmacol.* **2016**, *9*, 1533–1546, doi:10.1080/17512433.2016.1254041.
151. Jebabli, N.; Gaïes, E.; El Jebari, H.; Charfi, R.; Lakhal, M.; Klouz, A.; Trabelsi, S.; Salouage, I. Development and validation of a new HPLC method for determination of Lamotrigine and clinical application. *Tunis. Med.* **2015**, *93*, 565–568.
152. Baldelli, S.; Castoldi, S.; Charbe, N.; Cozzi, V.; Fucile, S.; Cattaneo, D.; Clementi, E. Comparison of the QMS Analyzer With HPLC-UV for the Quantification of Lamotrigine Concentrations in Human Plasma Samples: *Ther. Drug Monit.* **2015**, *37*, 689–694, doi:10.1097/FTD.0000000000000202.
153. Shaikh, A.S.; Li, W.; Yuan, G.; Gao, M.; Geng, C.; Guo, N.; Guo, R. Simple, rapid and highly sensitive HPLC method for measurement of Lamotrigine in human plasma and its clinical applications. *Pak. J. Pharm. Sci.* **2016**, *29*, 2245–2250.
154. Liu, L.; Zhao, L.; Wang, Q.; Qiu, F.; Wu, X.; Ma, Y. Influence of valproic acid concentration and polymorphism of UGT1A4*3, UGT2B7 -161C > T and UGT2B7*2 on serum concentration of lamotrigine in Chinese epileptic children. *Eur. J. Clin. Pharm.* **2015**, *71*, 1341–1347, doi:10.1007/s00228-015-1925-9.
155. Carlow, D.C.; Shi, H.; Schofield, R.C. Simultaneous Quantitation of Lamotrigine, Levetiracetam, 10-Hydroxycarbazepine, Topiramate, and Zonisamide in Serum Using HPLC-MS/MS. In *Clinical Applications of Mass Spectrometry in Drug Analysis*; Methods in Molecular Biology; Garg, U., Ed.; Humana Press: New York, NY, USA, 2016; Volume 1383, pp. 29–37, ISBN 978-1-4939-3251-1.
156. Domingues, D.S.; Pinto, M.A.L.; de Souza, I.D.; Hallak, J.E.C.; Crippa, J.A. de S.; Queiroz, M.E.C. Determination of Drugs in Plasma Samples by High-Performance Liquid Chromatography–Tandem Mass Spectrometry for Therapeutic Drug Monitoring of Schizophrenic Patients. *J. Anal. Toxicol* **2015**, *40*, 28-36, doi:10.1093/jat/bkv107.
157. Kwan, S.-Y.; Chuang, Y.-C.; Huang, C.-W.; Chen, T.-C.; Jou, S.-B.; Dash, A. Zonisamide: Review of Recent Clinical Evidence for Treatment of Epilepsy. *CNS Neurosci.* **2015**, *21*, 683–691, doi:10.1111/cns.12418.
158. Lourenço, D.; Sarraguça, M.; Alves, G.; Coutinho, P.; Araujo, A.R.T.S.; Rodrigues, M. A novel HPLC method for the determination of zonisamide in human plasma using microextraction by packed sorbent optimised by experimental design. *Anal. Methods* **2017**, *9*, 5910–5919, doi:10.1039/C7AY01912B.
159. Mohammadi, B.; Jalili, R.; Bahrami, G.; Majnooni, M. Rapid and sensitive high performance liquid chromatographic determination of zonisamide in human serum application to a pharmacokinetic study. *Indian J. Pharm. Sci.* **2012**, *74*, 360, doi:10.4103/0250-474X.107073.

160. Ibrahim, F.A.; El-Yazbi, A.F.; Wagih, M.M.; Barary, M.A. Chromatographic determination of zonisamide, topiramate and sulpiride in plasma by a fluorescent 'turn-on' chemosensor. *Bioanalysis* **2017**, *9*, 1049–1064, doi:10.4155/bio-2017-0090.
161. Villanueva, V.; Giráldez, B.G.; Toledo, M.; De Haan, G.J.; Cumbo, E.; Gambardella, A.; De Backer, M.; Joeres, L.; Brunnert, M.; Dedeken, P.; et al. Lacosamide monotherapy in clinical practice: A retrospective chart review. *Acta Neurol. Scand.* **2018**, *138*, 186–194, doi:10.1111/ane.12920.
162. Harris, J.A.; Murphy, J.A. Lacosamide and Epilepsy: Lacosamide and Epilepsy. *CNS Neurosci. Ther.* **2011**, *17*, 678–682, doi:10.1111/j.1755-5949.2010.00198.x.
163. Bauer, S.; Rudd, G.; Mylius, V.; Hamer, H.M.; Rosenow, F. Lacosamide intoxication in attempted suicide. *Epilepsy Behav.* **2010**, *17*, 549–551, doi:10.1016/j.yebeh.2010.01.007.
164. Greenaway, C.; Ratnaraj, N.; Sander, J.W.; Patsalos, P.N. A High-Performance Liquid Chromatography Assay to Monitor the New Antiepileptic Drug Lacosamide in Patients With Epilepsy. *Ther. Drug Monit.* **2010**, *32*, 448–452, doi:10.1097/FTD.0b013e3181dcc5fb.
165. Mouskeftara, T.; Alexandridou, A.; Krokos, A.; Gika, H.; Mastrogianni, O.; Orfanidis, A.; Raikos, N. A Simple Method for the Determination of Lacosamide in Blood by GC—MS. *J. Forensic Sci.* **2020**, *65*, 288–294, doi:10.1111/1556-4029.14151.
166. Korman, E.; Langman, L.J.; Jannetto, P.J. High-Throughput Method for the Quantification of Lacosamide in Serum Using Ultrafast SPE-MS/MS: *Ther. Drug Monit.* **2015**, *37*, 126–131, doi:10.1097/FTD.0000000000000115.
167. Nikolaou, P.; Papoutsis, I.; Spiliopoulou, C.; Voudris, C.; Athanaselis, S. A fully validated method for the determination of lacosamide in human plasma using gas chromatography with mass spectrometry: Application for therapeutic drug monitoring: Gas Chromatography. *J. Sep. Sci.* **2015**, *38*, 260–266, doi:10.1002/jssc.201400858.
168. De Liso, P.; Moavero, R.; Coppola, G.; Curatolo, P.; Cusmai, R.; De Sarro, G.; Franzoni, E.; Vigevano, F.; Verrotti, A. Current role of perampanel in pediatric epilepsy. *Ital. J. Pediatr.* **2017**, *43*, 51, doi:10.1186/s13052-017-0368-6.
169. Patsalos, P.N. The clinical pharmacology profile of the new antiepileptic drug perampanel: A novel noncompetitive AMPA receptor antagonist. *Epilepsia* **2015**, *56*, 12–27, doi:10.1111/epi.12865.
170. Mano, Y.; Takenaka, O.; Kusano, K. HPLC with fluorescence detection assay of perampanel, a novel AMPA receptor antagonist, in human plasma for clinical pharmacokinetic studies: Perampanel assay in human plasma by HPLC with fluorescence detector. *Biomed. Chromatogr.* **2015**, *29*, 1589–1593, doi:10.1002/bmc.3463.
171. Franco, V.; Marchiselli, R.; Fattore, C.; Tartara, E.; De Sarro, G.; Russo, E.; Perucca, E. Development and Validation of an HPLC-UV Assay for the Therapeutic Monitoring of the New Antiepileptic Drug Perampanel in Human Plasma: *Ther. Drug Monit.* **2016**, *38*, 744–750, doi:10.1097/FTD.0000000000000350.
172. Mohamed, S.; Candela, C.; Riva, R.; Contin, M. Simple and rapid validated HPLC-fluorescence determination of perampanel in the plasma of patients with epilepsy. *Pract. Lab. Med.* **2018**, *10*, 15–20, doi:10.1016/j.plabm.2017.11.003.
173. Mano, Y.; Takenaka, O.; Kusano, K. High-performance liquid chromatography–tandem mass spectrometry method for the determination of perampanel, a novel α-amino-3-hydroxy-5-methyl-4-isoxazolepropionic acid receptor antagonist in human plasma. *J. Pharm. Biomed. Anal.* **2015**, *107*, 56–62, doi:10.1016/j.jpba.2014.12.018.
174. Tůma, P.; Bursová, M.; Sommerová, B.; Horsley, R.; Čabala, R.; Hložek, T. Novel electrophoretic acetonitrile-based stacking for sensitive monitoring of the antiepileptic drug perampanel in human serum. *J. Pharm. Biomed. Anal.* **2018**, *160*, 368–373, doi:10.1016/j.jpba.2018.08.006.
175. Franco, V.; Baruffi, K.; Marchiselli, R.; Crema, F.; Fattore, C.; Romigi, A.; De Giorgis, V.; Tartara, E.; Elia, M.; D'Avolio, A.; et al. Determination of Perampanel in Dried Plasma Spots: Applicability to Therapeutic Drug Monitoring. *Ther. Drug Monit.* **2020**, *42*, 309–314, doi:10.1097/FTD.0000000000000680.
176. Steinhoff, B.J.; Hübers, E.; Kurth, C.; Jürges (Kehl-Kork), U. Plasma concentration and clinical effects of perampanel—The Kork experience. *Seizure* **2019**, *67*, 18–22, doi:10.1016/j.seizure.2019.02.022.
177. Mazzucchelli, I.; Rapetti, M.; Fattore, C.; Franco, V.; Gatti, G.; Perucca, E. Development and validation of an HPLC–UV detection assay for the determination of rufinamide in human plasma and saliva. *Anal. Bioanal. Chem.* **2011**, *401*, 1013–1021, doi:10.1007/s00216-011-5126-9.
178. Meirinho, S.; Rodrigues, M.; Fortuna, A.; Falcão, A.; Alves, G. Novel bioanalytical method for the quantification of rufinamide in mouse plasma and tissues using HPLC-UV: A tool to support pharmacokinetic studies. *J. Chromatogr. B* **2019**, *1124*, 340–348, doi:10.1016/j.jchromb.2019.06.021.

179. Gáll, Z.; Vancea, S.; Dogaru, M.T.; Szilágyi, T. Liquid chromatography–mass spectrometric determination of rufinamide in low volume plasma samples. *J. Chromatogr. B* **2013**, *940*, 42–46, doi:10.1016/j.jchromb.2013.07.014.
180. Contin, M.; Mohamed, S.; Candela, C.; Albani, F.; Riva, R.; Baruzzi, A. Simultaneous HPLC–UV analysis of rufinamide, zonisamide, lamotrigine, oxcarbazepine monohydroxy derivative and felbamate in deproteinized plasma of patients with epilepsy. *J. Chromatogr. B* **2010**, *878*, 461–465, doi:10.1016/j.jchromb.2009.11.039.
181. Dalvi, A.V.; Uppuluri, C.T.; Bommireddy, E.P.; Ravi, P.R. Design of experiments-based RP—HPLC bioanalytical method development for estimation of Rufinamide in rat plasma and brain and its application in pharmacokinetic study. *J. Chromatogr. B* **2018**, *1102–1103*, 74–82, doi:10.1016/j.jchromb.2018.10.014.
182. Fisher, J.L. The effects of stiripentol on GABAA receptors: Stiripentol and GABAA Receptors. *Epilepsia* **2011**, *52*, 76–78, doi:10.1111/j.1528-1167.2011.03008.x.
183. Eschbach, K.; Knupp, K.G. Stiripentol for the treatment of seizures in Dravet syndrome. *Expert Rev. Clin. Pharmacol.* **2019**, *12*, 379–388, doi:10.1080/17512433.2019.1605904.
184. Takahashi, R.; Imai, K.; Yamamoto, Y.; Takahashi, Y.; Hamano, S.; Yoshida, H. Determination of Stiripentol in Plasma by High-performance Liquid Chromatography with Fluorescence Detection. *Jpn. J. Pharm. Health Care Sci.* **2015**, *41*, 643–650, doi:10.5649/jjphcs.41.643.
185. Vossler, D.G. Remarkably High Efficacy of Cenobamate in Adults With Focal-Onset Seizures: A Double-Blind, Randomized, Placebo-Controlled Trial. *Epilepsy Curr* **2020**, *20*, 85–87, doi:10.1177/1535759720903032.
186. Oh, J.-H.; Jeong, J.-W.; Ji, Y.-G.; Shin, Y.-M.; Lee, K.-R.; Hyung Cho, K.; Koo, T.-S. Development of a liquid chromatography-tandem mass spectrometry method for assaying cenobamate in rat plasma. *J. Liq. Chromatogr. Relat. Technol.* **2018**, *41*, 992–997, doi:10.1080/10826076.2018.1547743.
187. Vernillet, L.; Greene, S.A.; Kim, H.W.; Melnick, S.M.; Glenn, K. Mass Balance, Metabolism, and Excretion of Cenobamate, a New Antiepileptic Drug, After a Single Oral Administration in Healthy Male Subjects. *Eur. J. Drug Metab. Pharm.* **2020**, *45*, 513–522, doi:10.1007/s13318-020-00615-7.
188. Patsalos, P.N.; Zugman, M.; Lake, C.; James, A.; Ratnaraj, N.; Sander, J.W. Serum protein binding of 25 antiepileptic drugs in a routine clinical setting: A comparison of free non-protein-bound concentrations. *Epilepsia* **2017**, *58*, 1234–1243, doi:10.1111/epi.13802.
189. Tuchila, C.; Baconi, D.; Pirvu, C.; Balalau, D.; Vlasceanu, A.; Stan, M.; Balalau, C. Therapeutic drug monitoring and methods of quantitation for carbamazepine. *J. Mind Med. Sci.* **2017**, *4*, 100–114, doi:10.22543/7674.42.P100114.
190. Raj Panday, D.; Panday, K.R.; Basnet, M.; Kafle, S.; Shah, B.; Rauniar, G. Therapeutic Drug Monitoring of Carbamazepine. *Int. J. Neurorehabilit. Eng.* **2017**, *4*, doi:10.4172/2376-0281.1000245.
191. Rosati, A.; De Masi, S.; Guerrini, R. Antiepileptic Drug Treatment in Children with Epilepsy. *CNS Drugs* **2015**, *29*, 847–863, doi:10.1007/s40263-015-0281-8.
192. Łuszczki, J.J. Third-generation antiepileptic drugs: Mechanisms of action, pharmacokinetics and interactions. *Pharmacol. Rep.* **2009**, *61*, 197–216, doi:10.1016/S1734-1140(09)70024-6.
193. Quintero, G.C. Review about gabapentin misuse, interactions, contraindications and side effects. *J. Exp. Pharmacol.* **2017**, *9*, 13–21, doi:10.2147/JEP.S124391.
194. Curia, G.; Biagini, G.; Perucca, E.; Avoli, M. Lacosamide: A New Approach to Target Voltage-Gated Sodium Currents in Epileptic Disorders. *CNS Drugs* **2009**, *23*, 555–568, doi:10.2165/00023210-200923070-00002.
195. Biton, V. Pharmacokinetics, toxicology and safety of lamotrigine in epilepsy. *Expert Opin. Drug Metab. Toxicol.* **2006**, *2*, 1009–1018, doi:10.1517/17425255.2.6.1009.
196. Zaccara, G.; Perucca, E. Interactions between antiepileptic drugs, and between antiepileptic drugs and other drugs. *Epileptic Disord.* **2014**, *16*, 409–431, doi:10.1684/epd.2014.0714.
197. Panebianco, M.; Prabhakar, H.; Marson, A.G. Rufinamide add-on therapy for refractory epilepsy. *Cochrane Database Syst. Rev.* **2018**, *4*, *CD011772*, doi:10.1002/14651858.CD011772.pub2.
198. Hsieh, D.T.; Thiele, E.A. Efficacy and safety of rufinamide in pediatric epilepsy. *Adv. Neurol. Disord.* **2013**, *6*, 189–198, doi:10.1177/1756285613481083.

199. Luszczki, J.J.; Ratnaraj, N.; Patsalos, P.N.; Czuczwar, S.J. Characterization of the Anticonvulsant, Behavioral and Pharmacokinetic Interaction Profiles of Stiripentol in Combination with Clonazepam, Ethosuximide, Phenobarbital, and Valproate Using Isobolographic Analysis. *Epilepsia* **2006**, *47*, 1841–1854, doi:10.1111/j.1528-1167.2006.00825.x.
200. Chiron, C. Stiripentol for the treatment of Dravet syndrome. *Orphan Drugs Res. Rev.* **2014**, *4*, 29–38, doi:10.2147/ODRR.S47619.
201. Buck, M.L.; Goodkin, H.P. Stiripentol: A Novel Antiseizure Medication for the Management of Dravet Syndrome. *Ann. Pharm.* **2019**, *53*, 1136–1144, doi:10.1177/1060028019856008.
202. Wang, X.; Ratnaraj, N.; Patsalos, P.N. The pharmacokinetic inter-relationship of tiagabine in blood, cerebrospinal fluid and brain extracellular fluid (frontal cortex and hippocampus). *Seizure* **2004**, *13*, 574–581, doi:10.1016/j.seizure.2004.01.007.
203. Ben-Menachem, E. Mechanism of action of vigabatrin: Correcting misperceptions: Vigabatrin mechanism of action. *Acta Neurol. Scand.* **2011**, *124*, 5–15, doi:10.1111/j.1600-0404.2011.01596.x.
